# Locomotor and endocrine alterations link to metabolic dysfunction induced by pathopharmacological interaction between neurodevelopmental disorders and antipsychotics: evidence from clinical and animal study

**DOI:** 10.3389/fpsyt.2026.1764492

**Published:** 2026-03-10

**Authors:** Menglu Zeng, Xinyu Yang, Zhenju Cao, Huiyu Chen, Yanfang Lu, Chen Xu, Danlin Weng, Anying Shen, Fei Xue, Wei Lin, Jianan Shi, Shuangyan Yang, Aifang Zhang, Fuchun Zhong, Yueqing Su

**Affiliations:** 1 Fujian Maternity and Child Health Hospital, College of Clinical Medicine for Obstetrics & Gynecology and Pediatrics, Fujian Medical University, Fuzhou, China; 2 Fujian Maternity and Child Health Hospital, Fuzhou, China; 3 School of Public Health, Fujian Medical University, Fuzhou, China; 4 Fuzhou First General Hospital, Affiliated with Fujian Medical University, Fuzhou, China

**Keywords:** adiponectin, insulin, leptin, locomotor activity, metabolic disturbance, neurodevelopmental disorders, Poly I:C, second-generation antipsychotic

## Abstract

**Background:**

Beyond the well-known metabolic side effects of second-generation antipsychotics (SGAs), recent studies suggest that neurodevelopmental disorders (NDDs) themselves confer an underlying susceptibility to metabolic dysregulation. However, it remains unclear whether a combined effect exists between SGAs and the NDD condition regarding metabolic syndrome, and which NDD-related pathophysiological changes contribute to metabolic disturbance.

**Methods:**

This study applies a translational framework combining retrospective clinical data from drug-naïve children with NDDs and a prenatal polyinosinic:polycytidylic acid (Poly I:C) rat model.

**Results:**

Baseline variations in lipid and glucose disturbances were observed in both cohorts, and these metabolic imbalances were further increased in adult female rats following long-term olanzapine or risperidone treatment, with significant interaction effect between Poly I:C and SGA observed for HOMA-IR. Moreover, the significant effects of both Poly I:C and SGAs on adipokines (leptin and adiponectin) and locomotor activity, with SGA-driven changes in insulin and prolactin, indicate that altered locomotion and divergent endocrine modulation serve as candidate pathways contributing to NDD-related metabolic risk.

**Discussion:**

These results highlight that NDD-related locomotor and endocrine changes should be considered as potential biological factors when finding effective strategies for preventing metabolic events during SGAs medication. These results underscore the clinical importance of metabolic monitoring in pediatric psychopharmacology, even prior to pharmacologic exposure.

## 1 Introduction

There is increasing clinical concern regarding metabolic disturbances in patients with neurodevelopmental disorders (NDDs) (e.g., autism spectrum disorders [ASD], attention-deficit/hyperactivity disorder [ADHD] and schizophrenia [SCZ]) due to their higher prevalence of metabolic syndrome, and elevated mortality resulting from cardiovascular diseases and diabetes compared to the general public (1–3). Emerging evidence indicates that the metabolic burden may evolve synchronously with neuropsychiatric symptoms, compounding health outcomes and reducing the quality of life in this population (4, 5). It remains unclear, however, whether these metabolic abnormalities represent an underlying susceptibility of the disease or are merely secondary to pharmacological treatment.

Second-generation antipsychotics (SGAs), notably olanzapine (OLZ) and risperidone (RISP), are widely prescribed for patients with NDDs (6, 7). While clinically effective, strong evidence has confirmed that SGAs are crucial risk factors for metabolic disturbance (8). Particularly in pediatric populations, SGAs are associated with significant weight gain and other tolerability issues, demanding rigorous safety monitoring (9). However, recent consensus has agreed that multiple risk factors contribute to metabolic disturbance, among which the pathophysiology of the disease itself is of particular interest (10). Indeed, clinical observations in first-episode or drug-naïve patients with NDDs have found dyslipidemia and impaired glucose tolerance accompanied by higher waist circumference and body mass index (BMI) (11), or a higher frequency of metabolic syndrome diagnoses and metabolic disturbances as a subsyndromal state (12, 13). Additionally, research suggests that metabolic syndrome and NDDs may share a common biological basis (14). However, despite these findings, limited preclinical studies have evaluated the contribution of NDDs themselves in metabolic disturbance during SGAs treatment (15, 16). Given the significant metabolic side effects of SGAs, it is worthwhile to explore whether there is a combined effect of SGAs and disease itself on the frequent metabolic syndrome complication in patients during SGAs medication, and which NDDs related pathophysiological changes heighten metabolic risk.

Abdominal obesity (AO), characterized by the accumulation of visceral and subcutaneous adipose tissues, plays a central role in the pathogenesis of metabolic syndrome (17). Weight gain due to energy intake exceeding energy expenditure is the major risk factor of AO (18). Studies have shown that children with NDDs exhibited significantly greater abdominal waist circumferences, with up to 60% of them having central obesity resulting from more energy intake (19–21). Notably, independent of SGA medication, increasing weight gain and appetite, as well as the change of locomotor patterns, are also observed in antipsychotic-naïve NDD patients (22–24). Moreover, since several hormones such as insulin, prolactin, leptin and adiponectin are recognized for their critical roles in metabolic regulation (25, 26), the concept of endocrine hormone disruption has recently been extended to metabolic alterations (27). Meanwhile, regarding endocrine hormones, changes in circulating prolactin, leptin, insulin, and adiponectin have also been shown in patients without medical treatment (14, 28–32). Therefore, it is reasonable to hypothesize that NDD-related locomotor changes and endocrine abnormalities may serve as candidate pathways contributing to the metabolic disturbance during SGAs treatment.

Epidemiological evidence demonstrates that gestational infection is an essential environmental risk factor of NDDs (33, 34), which is corroborated by findings in animal studies that maternal immune activation (MIA) alone is sufficient to impart lifelong neuropathological alterations and behavioral deficits in offspring (35). As a synthetic analog of double-stranded RNA viral mimetic agent for MIA, Polyinosinic: Polycytidylic acid (Poly I:C) is widely used to establish animal models for exploring the underlying pathogenesis of NDDs and identifying a new therapeutic strategy (36, 37). While historically linked to schizophrenia research, this model is increasingly utilized to investigate shared neurobiological vulnerabilities, such as neuroinflammation and metabolic dysregulation that are common across the NDD spectrum, including ASD and ADHD (38, 39). Despite observed sex differences in rodent MIA models for NDD, preclinical studies are predominantly male focused to avoid variability introduced by estrogens (40, 41). Our previous work has confirmed that prenatal Poly I:C exposure results in behavioral deficits in female adolescent offspring, and SGAs administration in male juvenile offspring exerts long-lasting effects on adult behaviors (42, 43). Nonetheless, the baseline metabolic profile (e.g., glucose and lipids) in both sexes during the critical early developmental stages has yet to be fully assessed. Therefore, to evaluate the hypothesis of disease-related vulnerability and susceptibility to metabolic syndrome during SGAs medication, in this study, we first assessed lipids and glucose metabolism in both drug-naïve NDDs patients (ASD and ADHD) and an etiologically relevant rat model induced by Poly I:C to obtain baseline metabolic changes. To further investigate the heightened risk of SGA-induced metabolic dysregulation in females, we focused on an adult female rat model receiving five weeks of olanzapine or risperidone treatment. In addition to basic serum biochemical parameters for both lipid and glucose profile, the effect of prenatal Poly I:C challenge and/or chronic SGAs treatment on locomotor patterns, endocrine hormones of prolactin, adiponectin, leptin, and insulin, as well as the risk factors which have been reported to be associated with metabolic dysfunction were assessed, including weight gain, fat accumulation and appetite.

## 2 Materials and methods

### 2.1 Ethical statements

The clinical study was approved by the Institutional Medical Ethics Review Board of Fujian Maternity and Child Health Hospital (Approval NO. 2024KY141), and the requirement for informed consent was waived due to the retrospective design. Animal experimental procedures were approved by the Animal Ethics Committee, Fujian Maternity and Child Health Hospital, Fujian, China (AEC SFY 2025 013).

### 2.2 Clinical study

This retrospective study identified patients from the electronic medical records of Fujian Maternity and Child Health Hospital (Fuzhou, China) from January 2021 to December 2024. A total of 234 patients with NDDs [152 children with ADHD (male = 129, female = 23) and 82 children with ASD (male = 68, female = 14)] aged 3–13 years and 191 healthy controls (male = 146, female = 45) were enrolled in this study. The inclusion criteria for NDD patients were: (1) a confirmed diagnosis of ASD or ADHD by an experienced child psychiatrist according to the Diagnostic and Statistical Manual of Mental Disorders, Fifth Edition (DSM-5); (2) aged between 3 and 13 years; (3) no documented history of antipsychotic medication use; and (4) available and complete results for laboratory data. Healthy controls were identified from routine pediatric visits for health screenings in the same hospital department during the same period. The inclusion criteria for controls were: (1) aged between 3 and 13 years; (2) no documented neurodevelopmental or psychiatric diagnoses; and (3) available and complete results for laboratory data. To ensure group comparability and minimize selection bias, exclusion criteria were applied symmetrically to both the NDD and control groups. Participants were excluded if they had: (1) epilepsy or identifiable genetic syndromes (Trisomy 21, Fragile X syndrome, Rett syndrome, etc.); (2) pre-existing inherited metabolic diseases or severe systemic infections/inflammation; and (3) malignant tumors or other serious physical illnesses. For all participants, in addition to metabolic profiles (lipids and glucose), demographic and anthropometric data including age, sex, height, and weight were recorded to calculate BMI. The time of blood draw (categorized as AM/PM) was also recorded to account for potential variations in fasting status during statistical adjustments.

### 2.3 Animal study

#### 2.3.1 Experimental design

**Figure 1:**
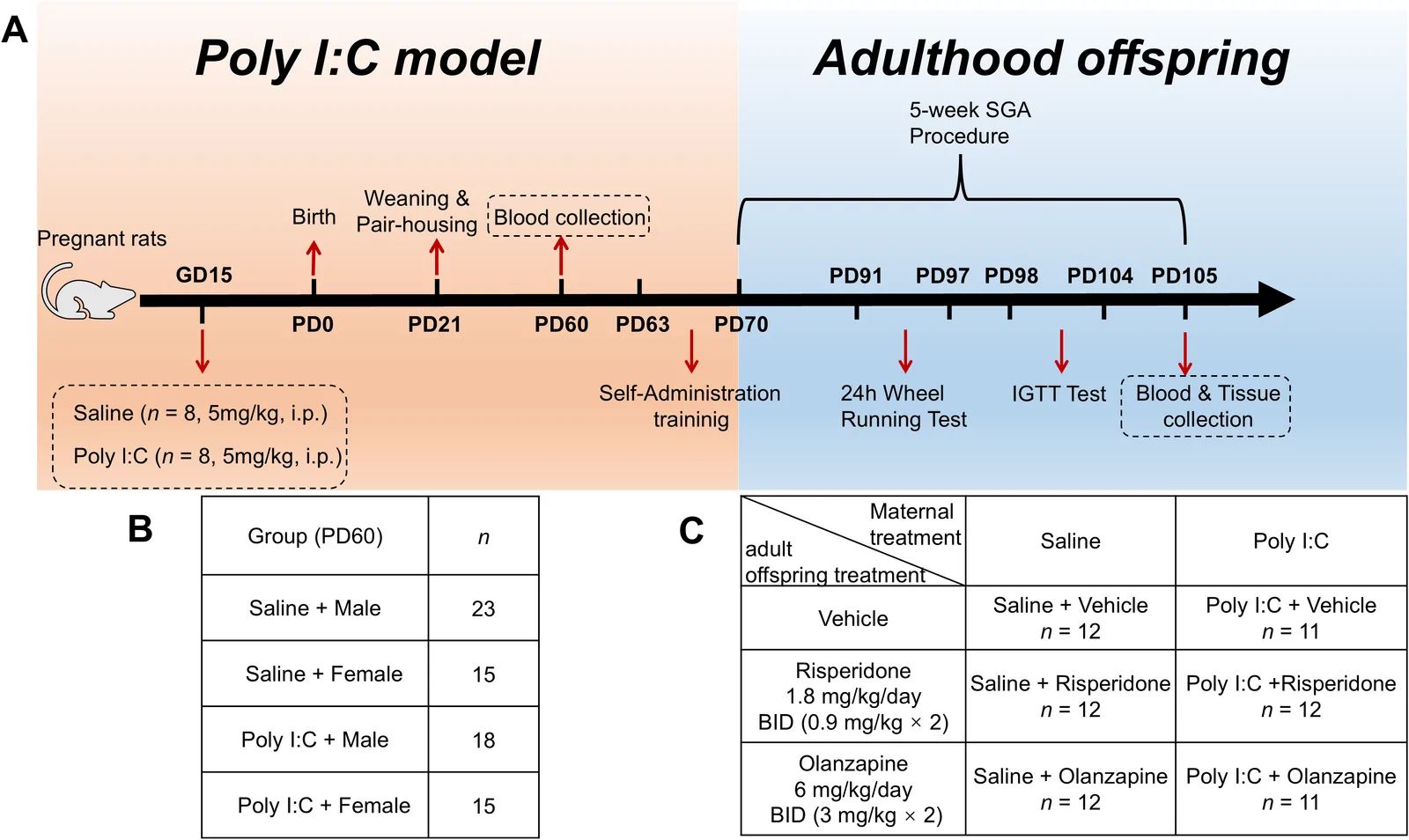
Experimental design and animal grouping. **(A)** Timeline of the animal experiment. **(B)** Experimental grouping at PD60. **(C)** Experimental grouping at PD70. Poly I:C, Polyriboinosinic-Polyribocytidylic acid; i.p., intraperitoneal; GD, gestation day; PD, postnatal day; IGTT, intraperitoneal glucose tolerance test; SGAs, second-generation antipsychotics.

Animal experiments were conducted as shown in **Figure 1A**. The method for constructing the NDD rat model by prenatal Poly I:C-exposure was described in our previous work, resulting in NDD-like behavioral impairments in both adolescent and adult offspring (42–44). In brief, 16 pregnant Sprague–Dawley rats were obtained at gestation day (GD) 8 from the Sipaifu Biotechnology Co., LTD. (Beijing, China). At GD15 (mid-late gestation), they were separated into 2 groups randomly (*n* = 8). One group received an intraperitoneal (IP) injection of 5 mg/kg Poly I:C (Poly I:C-Low Molecular Weight; Invivogen, Toulouse, France) dissolved in 0.2 ml 1% phosphate buffered saline (PBS)/20 g, while the other group received an equivalent volume of PBS. Day of birth was considered postnatal day (PD) 0. Pups were weaned and pair-housed according to sex at PD21. To minimize litter effects (pseudoreplication), typically 1–2 (maximum 3) pups per sex were selected from each litter (**Supplementary Table 6**). At PD60, adolescent offspring from four randomly selected dams in each group (*n* = 71, grouping details shown in **Figure 1B**) were used for blood collection to assess metabolic parameters. To further investigate whether and how metabolic disturbances were influenced by long-term treatment with SGAs, all female offspring (*n* = 70) were selected for the study, considering that estrogen-mediated enhancement of dopamine sensitivity renders females more prone to SGA-induced adverse effects (45). While all male offspring were assigned to a separate study with a different design (data not shown).

At PD 70, the 36 Saline and 34 Poly I:C female offspring were randomly divided into 3 subgroups (*n* = 11-12/group; **Figure 1C**): Saline + Vehicle (Veh), Saline + OLZ, Saline + RISP, Poly I:C + Veh, Poly I:C + OLZ, Poly I:C + RISP. Drug treatment lasted for five weeks (twice daily at 08:00h and 18:00h). Dosages were set at 6 mg/kg/day for OLZ and 1.8 mg/kg/day for RISP. These specific dosages were selected based on translational pharmacokinetics. Due to the significantly faster metabolism of SGAs in rodents, these doses achieve plasma exposure levels and D2 receptor occupancy levels equivalent to therapeutic ranges in clinical pediatric settings (46–49).

Prior to drug treatment, rats were trained to self-administer a sweet cookie dough pellet (0.3 g) without drugs twice a day for one week. The pellet was made of 36% corn flour, 36% sugar, 20% gelatine, and 8% milk powder with deionized water. To ensure precise dosing and stability, the clinically used drug tablets were ground into fine powder, weighed, and mixed with droplets of water, immediately prior to administration to ensure maximal drug stability and bioactivity. The pellets with or without drugs were then offered to rats on a metal spoon and observed to ensure complete consumption by the rats. To ensure standardized drug exposure over time, body weight was recorded weekly during the five weeks of drug treatment, and the specific drug content in the pellets for each rat was recalculated based on body weight every week and incorporated into a fixed mass of cookie dough (0.3 g). In addition to body weight, food intake and water intake of rats were also measured once a week during the five weeks of drug treatment. Body length was measured using a vernier caliper.

In order to observe the influence of SGA administration and prenatal Poly I:C challenges on locomotor style and activity, a 24-hour running wheel test was performed during the fourth week of drug treatment (PD 91-97). In the fifth week of drug treatment (PD 98-104), an IGTT was conducted. At PD 105, rats were fasted overnight and received their final dose, and sacrificed 2 hours later under isoflurane anesthesia for blood and tissue collection. This time point was chosen because the half-lives of OLZ (~2.5 h) and RISP (~1 h) in rats were significantly shorter than in humans (46, 50). Sampling at 2 hours aligned with the peak steady-state plasma concentration and maximal receptor occupancy, ensuring the metabolic profile reflected active pharmacological engagement (46, 50).

All animals were allowed ad libitum access to water and standard laboratory chow diet (3.9 kcal/g: 10% fat, 74% carbohydrate, 16% protein) under environmentally controlled conditions (22°C, light cycle from 07:00h to 19:00h and dark cycle from 19:00h to 07:00h) throughout the experimental period.

#### 2.3.2 Twenty-four-hour voluntary wheel running test

Following 5 consecutive days of wheel running training (10min/day), each rat was put into an unlocked wheel for 48 hours and their voluntary running distance (cm) was recorded every 30 min. Data from the first 24 hours were excluded as habituation, and data from the second 24-hour period were analyzed using a computer and Vital View Software System (Philips Respironics, Pittsburgh, USA).

#### 2.3.3 Intraperitoneal glucose tolerance test

Following an overnight fast and 2 hours of drug treatment, rats were administered glucose (2 g/kg body weight) via IP injection. Meanwhile, blood samples from the tail vein were drawn to evaluate the glucose levels at 0, 15, 30, 60, 90, 120, and 180 min post-injection using an automatic glucometer (ACCU-CHEK mobile, Roche Diabetes Care, North Ryde, NSW, Australia). The area under the glucose curve (AUC) over 0–180 min was calculated for each animal using the trapezoidal rule.

#### 2.3.4 Blood and tissue collection

Blood was collected from the tail vein under isoflurane anesthesia and allowed to clot at room temperature for 15–30 min. Blood samples were then centrifuged at 2,000 × g for 10 min at 4 °C, and the serum was collected and stored at −80 °C until biochemical analysis. Perirenal, periovarian, mesenteric, and inguinal white fat pads were dissected and weighed individually, and their combined weight was recorded as total white fat.

### 2.4 Blood metabolic parameters analysis

#### 2.4.1 Biochemical analysis for metabolic parameters

Meikang commercial Kits (Meikang Biotechnology, Ningbo, China) were used to determine the concentration of triglyceride (TG), total cholesterol (TC), high-density lipoprotein cholesterol (HDL-c), low-density lipoprotein cholesterol (LDL-c), Apolipoprotein A-I (ApoA-I) and Apolipoprotein B (ApoB) in the serum using an ARCHITECT Ci16200 analyzer (Abbott Laboratories Ltd, Abbott Park, Illinois, US).

#### 2.4.2 Enzyme-linked immunosorbent assay for hormones

Commercial ELISA kits were used to quantify the concentrations of insulin (Merck Millipore, Billerica, USA), as well as prolactin, adiponectin and leptin (Demeditec Diagnostics GmbH, Kiel, Germany) in the serum in strict accordance with the manufacturers’ protocols. Insulin resistance was calculated using the homeostasis model assessment (HOMA-IR: fasting glucose (mmol/L) **×** fasting insulin (mU/L)/22.5).

### 2.5 Statistical analysis

All data were analyzed using SPSS 25.0 (IBM, New York, USA) and visualized with GraphPad Prism 9.5.0 (San Diego, California, USA). The validity of statistical assumptions for both General Linear Models (GLM) and Linear Mixed Models (LMM) was rigorously verified. Normality was assessed via Shapiro-Wilk tests, model residuals were inspected using Normal Q-Q plots and histograms, and homogeneity of variances was evaluated with Levene’s test. Non-normally distributed variables were naturally log-transformed (ln) to meet model assumptions. Outliers were identified using the ROUT method (Q = 1%); primary analyses were conducted without outlier removal, while sensitivity analyses excluding ROUT-flagged values were performed to verify the robustness (presented in **Supplementary Tables 4**, **5**, **7–10**). Group comparisons and effect estimates were based on Estimated Marginal Means (EMMs). Clinical data were analyzed using a two-way analysis of covariance (ANCOVA) within the GLM, with Group (Control, ASD, ADHD) and Sex (Male, Female) and Group × Sex included as fixed factors, adjusting for Age, BMI and time of blood draw as covariates. EMMs were computed at the sample mean values of covariates. *Post-hoc* comparisons were performed with Bonferroni correction. For each effect, *F*-statistics with degrees of freedom, *p*-value, and partial eta-squared (*η*²) were reported. Pairwise comparisons are reported as mean differences (95% CIs) for untransformed variables. For log-transformed variables, differences in EMMs were back-transformed and reported as Geometric Mean Ratios (GMRs) with 95% CIs, representing the relative fold-change between groups. Animal data, except IGTT, were analyzed using an LMM with Prenatal condition (Saline, Poly I:C), Drug (Vehicle, OLZ, RISP), and their interaction as fixed factors, and litter included as a random effect, followed by Sidak adjustment. IGTT data were analyzed using a mixed model for repeated measures (MMRM). Fixed effects included prenatal condition (Saline, Poly I:C), drug treatment (Vehicle, OLZ, RISP), time, and all interaction terms. Type III tests of fixed effects were reported. Litter (Dam ID) was modeled as a random intercept (variance components) to account for within-litter clustering, and within-animal repeated measurements over time were modeled at the animal level (Rat ID nested within Dam ID) using a first-order autoregressive covariance structure [AR(1)]. Models were fitted using restricted maximum likelihood (REML). *Post hoc* comparisons were conducted using EMMs from the Prenatal × Drug × Time model, with Sidak adjustment. The Pearson correlation test was used to determine the relationships between the various measurements. All tests were two-tailed, and a *p*-value < 0.05 was considered statistically significant. For graphical presentation, EMMs and their corresponding standard errors (SEMs) were back-transformed to the original scale.

## 3 Result

### 3.1 Metabolic abnormalities in young NDDs

#### 3.1.1 Clinical study

The demographic and clinical characteristics of the study participants are summarized in **Table 1**.

**Table 1:** Demographic and clinical characteristics of the study participants.

Characteristics	Control (n=191)	ASD (n=82)	ADHD (n=152)
Age, IQR	6 [5,8]	8 [7,10]	8 [7,10]
BMI, IQR	15.4 [14.5,16.8]	15.6 [14.6,17.7]	16 [14.9,17.1]
Sex, n(%)			
Male	146 (76.4%)	68 (82.9%)	129 (84.9%)
Female	45 (23.6%)	14 (17.1%)	23 (15.1%)
Time of blood draw, n(%)			
Morning	168 (88.0%)	69 (84.1%)	137 (90.1%)
Afternoon	23 (12.0%)	13 (15.9%)	15 (9.9%)

Continuous variables are presented as median [interquartile range (IQR)], and categorical variables as n (%). This table is descriptive; primary inference is based on covariate−adjusted generalized linear models (GLMs) that included age, BMI, and time of blood draw (AM/PM) as covariates (see Methods and **Supplementary Material**). Time of blood draw was recorded only as morning (AM) versus afternoon (PM); exact clock time, fasting status, and pubertal status were not available. Obesity was not an exclusion criterion; BMI distributions confirm that individuals with obesity were present across all groups, including controls. Individuals with documented metabolic diseases were not enrolled based on medical history screening; however, undiagnosed or unrecorded metabolic conditions cannot be definitively ruled out.

##### 3.1.1.1 Lipid metabolism

**Figure 2:**
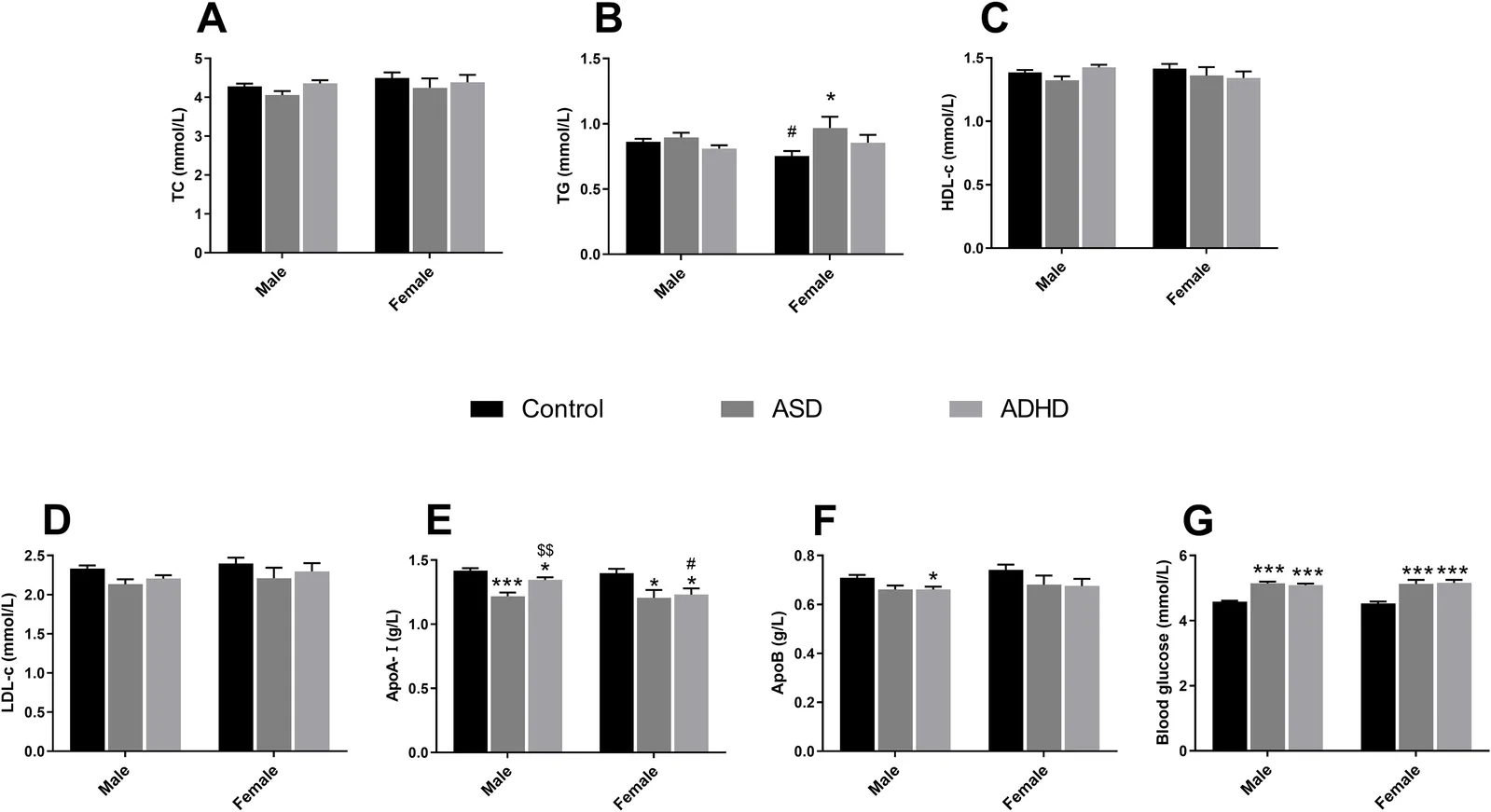
Biochemical metabolic parameters in children with NDDs. Panels show serum levels of **(A)** TC, **(B)** TG, **(C)** HDL-c, **(D)** LDL-c, **(E)** ApoA-I, **(F)** ApoB, and **(G)** Glucose. Data are presented as EMMs ± SEM. For variables analyzed using natural log-transformation, values are presented as back-transformed geometric means, and SEMs were approximated using the delta method (or derived from back-transformed confidence intervals) to maintain visual consistency. Sample sizes: males (Control, *n* = 146; ASD, *n* = 68; ADHD, *n* = 129) and females (Control, *n* = 45; ASD, *n* = 14; ADHD, *n* = 23). ^*^p < 0.05, ^***^p < 0.001 *vs* Control within the same sex; ^#^*p* < 0.05 *vs* males within the same group; ^$$^*p* < 0.01 *vs* ASD within the same sex. NDDs: Neurodevelopmental disorders. ASD, autism spectrum disorder; ADHD, attention-deficit/hyperactivity disorder; TC, total cholesterol; TG, triglyceride; HDL-c, high-density lipoprotein cholesterol; LDL-c, low-density lipoprotein cholesterol; ApoA-I, apolipoprotein A-I; ApoB, apolipoprotein B; EMM, estimated marginal mean; SEM, Standard Error of the Mean.

TG levels were natural log-transformed (Ln_TG). After adjusting for significant covariates (Age, BMI and time of blood draw; all *p* < 0.01), the GLM revealed a significant main effect of Group [*F*(2, 416) = 3.041, *p* = 0.049, partial *η*² = 0.014] and a marginally significant interaction effect between Group and Sex [*F*(2, 416) = 2.812, *p* = 0.061, partial *η*² = 0.013; **Figure 2B**]. *Post-hoc* tests displayed that TG levels were significantly higher in ASD group compared to the Control group (GMR = 1.283, 95% CI: 1.001-1.642, *p* = 0.049) in female cohort. While in Control group, males exhibited significantly higher TG levels than females (GMR = 1.142, 95% CI: 1.020–1.278, *p* = 0.021). For ApoA-I, the covariates (Age, BMI and time of blood draw) did not show significant effects (all *p* > 0.05), while the ANCOVA revealed significant main effects of Group [*F*(2, 416) = 14.357, *p* < 0.001, partial *η*² = 0.065; **Figure 2E**]. The main effects of Sex (*p* = 0.117) and the Group × Sex interaction (*p* = 0.303) was not statistically significant. In males, significant differences were found across all three groups. The ASD group showed the lowest ApoA-I levels, which were significantly lower than both the Control group (Mean Difference = -0.200, 95% CI: -0.283 to -0.118, *p* < 0.001) and the ADHD group (Mean Difference = -0.128, 95% CI: -0.212 to -0.044, *p* = 0.001). The ADHD group also showed significantly reduced levels compared to controls (Mean Difference = -0.073, 95% CI: -0.139 to -0.006, *p* = 0.027). In females, a similar reduction in clinical groups was observed. Both ASD patients (Mean Difference = -0.191, 95% CI: -0.362 to -0.019, *p* = 0.023) and ADHD patients (Mean Difference = -0.165, 95% CI: -0.307 to -0.024, *p* = 0.016) had significantly lower ApoA-I levels compared to Controls. After adjusting for significant covariates (Age, BMI and time of blood draw; all *p* < 0.001), there was a significant main effect of Group on ApoB [*F*(2, 416) = 5.210, *p* = 0.006, partial *η*² = 0.024; **Figure 2F**], while the main effect of Sex (*p* = 0.236) and the Group × Sex interaction (*p* = 0.902) were not statistically significant. In males, ApoB levels were significantly lower in the ADHD group compared to controls (Mean Difference = -0.049, 95% CI: -0.090 to -0.009, *p* = 0.011), with a similar marginally significant trend observed in ASD males (Mean Difference = -0.048, 95% CI: -0.098 to 0.002, *p* = 0.063). In females, although comparisons did not reach statistical significance, clinical groups exhibited mean reductions in ApoB levels comparable to those seen in males. Specifically, the mean difference relative to controls was -0.061 for ASD females (95% CI: -0.165 to 0.044, *p* = 0.488) and -0.066 for ADHD females (95% CI: -0.152 to 0.020, *p* = 0.194). For the remaining lipids, there were no significant main effects of Group or Sex, as well as interaction effects of these two factors on TC, HDL-c and LDL-c (*p* > 0.05; **Figures 2A, C, D**).

These results indicated that drug-naïve patients with NDDs exhibited altered lipid profiles (after adjusting for covariates), specifically characterized by decreased ApoA-I and ApoB levels across clinical groups and a potential sex-specific elevation of TG in females with ASD.

##### 3.1.1.2 Glucose metabolism

Glucose (GLU) levels were log-transformed (Ln_GLU).After adjustment for covariates (time of blood draw: *p* = 0.024; Age and BMI: *p* > 0.05), the GLM revealed a significant main effect of Group [*F*(2, 416) = 58.038, *p* < 0.001, partial *η*² = 0.218; **Figure 2G**]. No significant Sex × Group interaction was found (*p* > 0.05). *Post-hoc* comparisons showed that GLU levels were significantly elevated in both clinical groups compared to controls, with consistent trends across sexes. Specifically, in male cohort, both ASD and ADHD groups exhibited significantly higher GLU levels compared to healthy controls (ASD *vs* Control: GMR = 1.122, 95% CI: 1.088-1.158, *p* < 0.001; ADHD *vs* Control: GMR = 1.112, 95% CI: 1.083-1.141, *p* < 0.001). Similarly, in females, ASD and ADHD groups also exhibited significantly higher GLU levels compared with controls, (ASD *vs* Control: GMR = 1.134, 95% CI: 1.062-1.210, *p* < 0.001; ADHD *vs* Control: GMR = 1.140, 95% CI: 1.080-1.203, *p* < 0.001), suggesting a tendency toward impaired glucose metabolism.

**Supplementary Tables 1**–**3** presented the summary of covariate−adjusted GLMs for clinical metabolic outcomes, EMMs (95% CI) of clinical metabolic outcomes, Bonferroni−adjusted pairwise comparisons of EMMs for each clinical metabolic outcome. Sensitivity analyses excluding the identified outliers (**Supplementary Table 4**) showed results consistent with the primary analysis (**Supplementary Table 5**), except that the main Group effect on LDL-c shifted from marginal significance (*p* = 0.059) to statistical significance (*p* = 0.047), confirming the robustness of the clinical findings.

#### 3.1.2 Animal study

##### 3.1.2.1 Lipid metabolism

**Figure 3:**
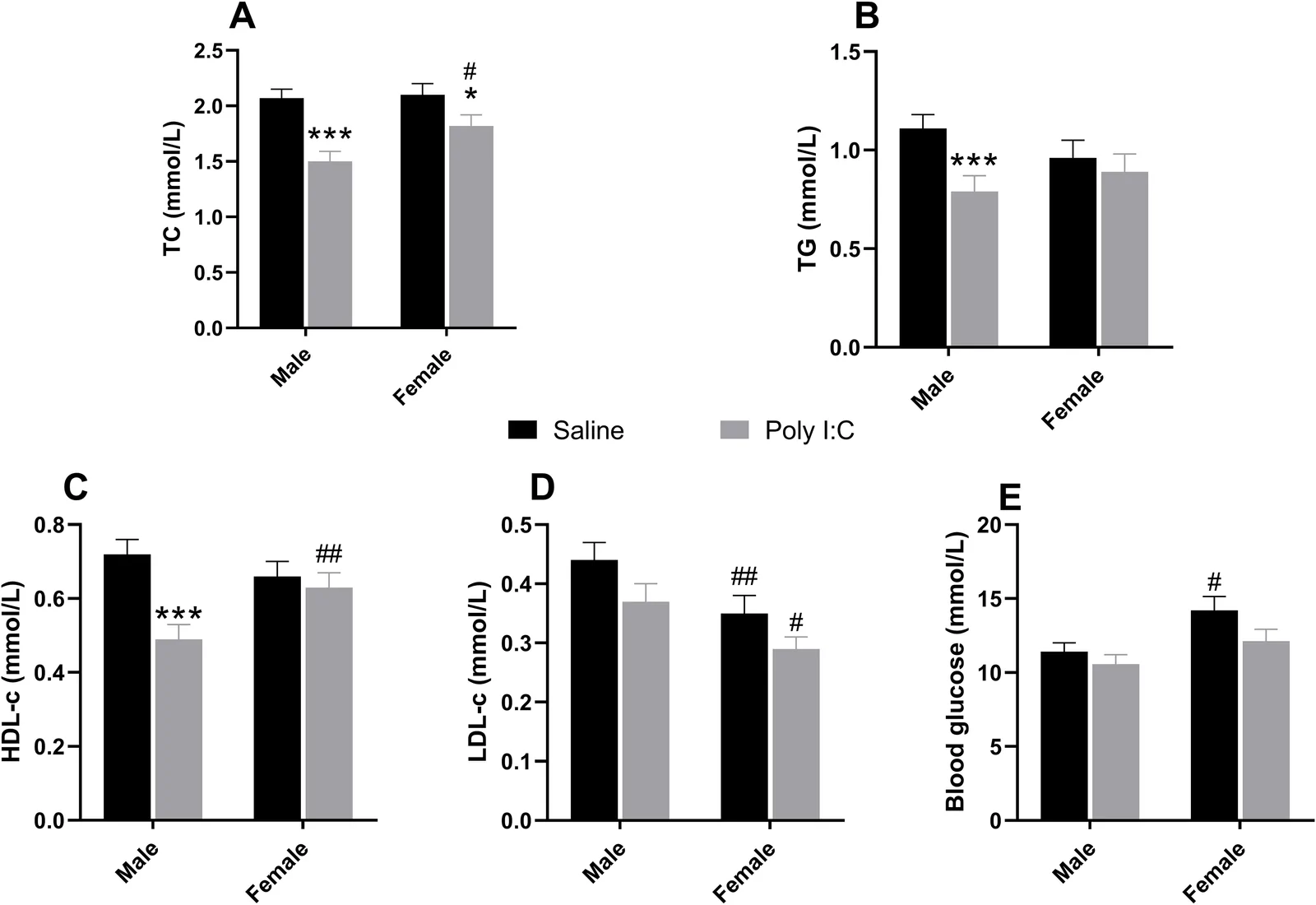
Biochemical metabolic parameters in juvenile Poly I:C offspring. Panels show serum levels of **(A)** TC, **(B)** TG, **(C)** HDL-c, **(D)** LDL-c, and **(E)** Glucose. Data are presented as EMMs ± SEM. For variables analyzed using natural log-transformation, values are presented as back-transformed geometric means, and SEMs were approximated using the delta method (or derived from back-transformed confidence intervals) to maintain visual consistency. Sample sizes: females (*n* = 15 per group); males (Saline, *n* = 23; Poly I:C, *n* = 18). ^*^p < 0.05, ^***^p < 0.001 *vs* Saline within the same sex; ^#^*p* < 0.05, ^##^*p* < 0.01 *vs* another sex within the same prenatal treatment. TC, total cholesterol; TG, triglyceride; HDL-c, high-density lipoprotein cholesterol; LDL-c, low-density lipoprotein cholesterol; Poly I:C, Polyriboinosinic-Polyribocytidylic acid (5 mg/kg, at GD 15); EMM, estimated marginal mean; SEM, Standard Error of the Mean.

For TC levels, LMM analysis identified significant main effects of both prenatal Poly I:C exposure [*F*(1, 14.1) = 15.410, *p* = 0.002] and sex [*F*(1, 53.5) = 5.730, *p* = 0.020]. No significant interaction effect was observed (*p* > 0.05). *Post-hoc* analysis showed that Poly I:C exposure significantly reduced TC levels in both male (*p* < 0.001 *vs* Saline + Male) and female (*p* = 0.045 *vs* Saline + Female) offspring. Furthermore, the TC levels in Poly I:C + Male group were considerably lower than Poly I:C + Female group (*p* = 0.004; **Figure 3A**). For TG concentrations, only a significant main effect of Poly I:C factor was observed [*F*(1, 13.8) = 5.190, *p* = 0.039], while the Sex × Poly I:C interaction was not statistically significant (*p* = 0.099). Poly I:C also distinctly decreased TG levels in male offspring (*p* = 0.005 *vs* Saline + Male; **Figure 3B**). For HDL-c levels, a significant main effect of Poly I:C [*F*(1, 14.3) = 7.120, *p* = 0.018] and a significant interaction effect between Poly I:C and sex [*F*(1, 53.6) = 10.300, *p* = 0.002] were observed. Similarly, Poly I:C treatment substantially reduced HDL-c levels in male offspring (*p* < 0.001 *vs* Saline + Male). Besides, the HDL-c levels in Poly I:C + Male group were prominently lower than Poly I:C + Female group (*p* = 0.003; **Figure 3C**). For LDL-c levels, there was a significant main effect of sex [*F*(1, 53.7) = 14.160, *p* < 0.001]. *Post-hoc* analysis revealed that female rats exhibited lower LDL-c levels in both Saline group (*p* = 0.008 *vs* Saline + Male) and Poly I:C group (*p* = 0.014 *vs* Poly I:C + Male; **Figure 3D**). These findings suggested that drug-naïve rodents models of NDDs exhibited distinct lipid metabolic alterations, characterized by a hypolipidemic profile with reduced TC, TG, and HDL-c levels, particularly in male offspring.

##### 3.1.2.2 Glucose metabolism

There was only a significant main effect of sex on blood glucose [*F*(1, 67.0) = 8.410, *p* = 0.005], but no significant effect of prenatal Poly I:C exposure or Sex × Poly I:C interaction was found (*p* = 0.061, *p* = 0.506, respectively). Notably, the GLU levels were prominently higher in female saline rats than male saline rats (*p* = 0.012; **Figure 3E**).

**Supplementary Table 7** showed linear mixed model results for PD60 metabolic outcomes. The drug-naïve metabolic patterns of rat at PD60 remained stable after excluding outliers, with no significant changes observed in the overall statistical conclusions (**Supplementary Tables 8**, **9**).

Collectively, drug-naïve patients and the Poly I:C rat model displayed distinct metabolic profiles. While patients showed elevated TG in females with ASD and reduced ApoA-I and ApoB, the animal model displayed a male-specific hypolipidemic profile without replicating human glucose changes. These findings highlighted the influence of biological sex and suggested species-specific metabolic vulnerabilities.

### 3.2 Effect of SGAs and/or Poly I:C on metabolism in adult offspring

#### 3.2.1 Lipid metabolism

**Figure 4:**
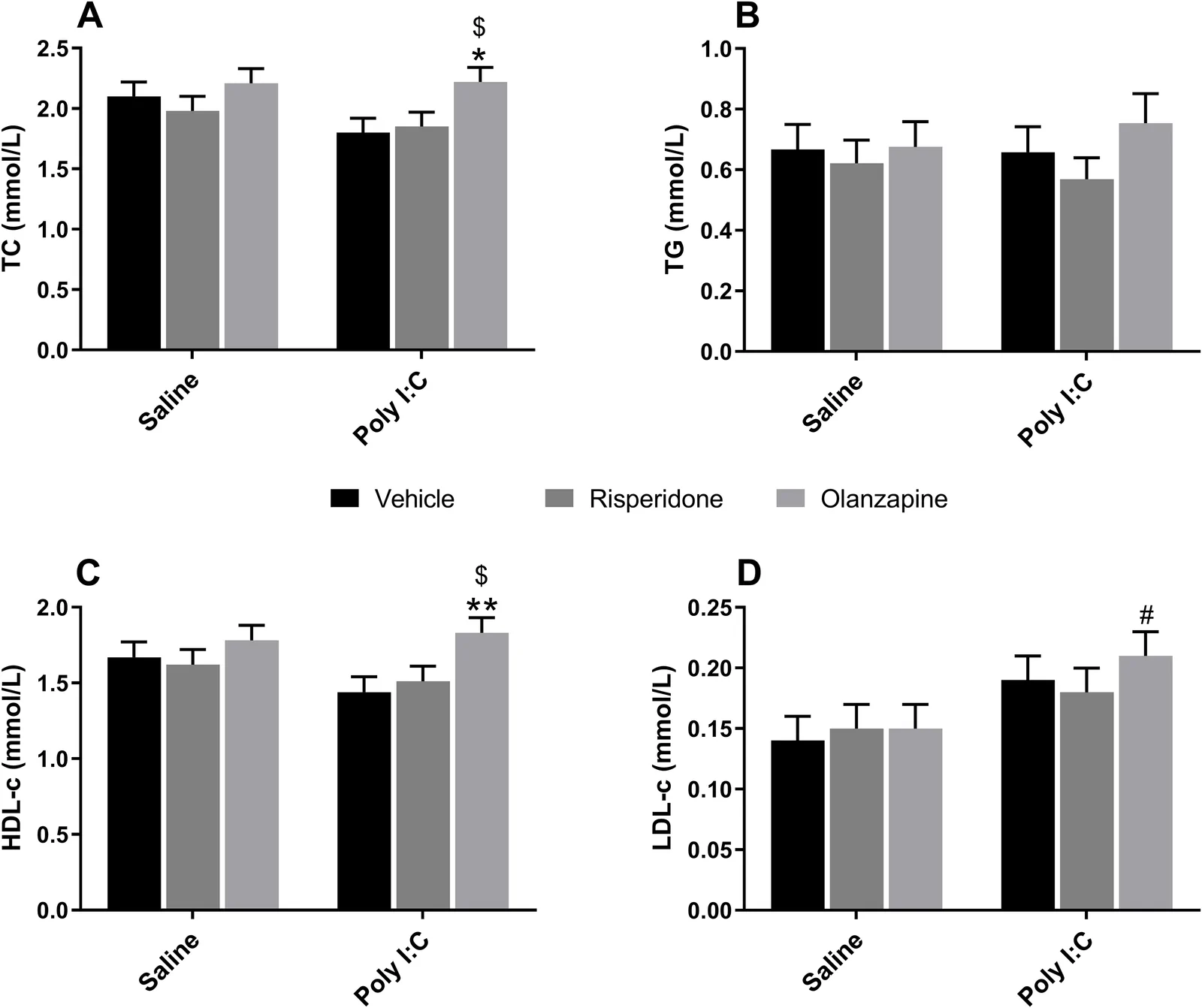
Effect of long-term SGA treatment and/or prenatal Poly I:C exposure on lipid metabolism in female adult offspring. Panels show serum levels of **(A)** TC, **(B)** TG, **(C)** HDL-c, and **(D)** LDL-c. Data are presented as EMMs ± SEM. For variables analyzed using natural log-transformation, values are presented as back-transformed geometric means, and SEMs were approximated using the delta method (or derived from back-transformed confidence intervals) to maintain visual consistency. Sample sizes: *n* = 12 per group, except for Poly I:C + OLZ and Poly I:C + Veh groups (both *n* = 11). ^*^p < 0.05, ^**^p < 0.01 *vs* Poly I:C + Veh; ^#^*p* < 0.05 *vs* Saline + OLZ; ^$^*p* < 0.05 *vs* Poly I:C + RISP. Poly I:C, Polyriboinosinic-Polyribocytidylic acid (5 mg/kg, at GD 15); TC, total cholesterol; TG, triglyceride; HDL-c, high-density lipoprotein cholesterol; LDL-c, low-density lipoprotein cholesterol. Veh, vehicle; OLZ, olanzapine; RISP, risperidone; EMM, estimated marginal mean; SEM, Standard Error of the Mean.

LMM analysis revealed significant main effects of SGAs on TC [*F*(2, 50.0) = 5.630, *p* = 0.006] and HDL-c levels [*F*(2, 50.0) = 5.390, *p* = 0.008], along with a significant main effect of Poly I:C on LDL-c levels [*F*(1, 12.7) = 6.170, *p* = 0.028] (**Figures 4A, C, D**). No significant Poly I:C × Drug interactions were observed for TC, HDL-c, and LDL-c levels (all *p* > 0.05). Similarly, no significant main effects or interactions were found for TG levels (all *p* > 0.05; **Figure 4B**). *Post-hoc* analysis showed that OLZ treatment significantly increased TC level in Poly I:C-exposed rats compared to both the RISP (*p* = 0.034) and vehicle groups (*p* = 0.013; **Figure 4A**). Similarly, OLZ-treated Poly I:C rats exhibited higher HDL-c levels than those receiving RISP (*p* = 0.031) or vehicle (*p* = 0.009) treatment (**Figure 4C**). Besides, LDL-c levels were significantly higher in Poly I:C rats treated with OLZ compared to Saline rats with the same treatment (*p* = 0.030; **Figure 4D**). These results suggested that while both SGA administration and prenatal Poly I:C exposure independently influenced serum lipids, their combined impact did not reflect as a synergistic interaction.

#### 3.2.2 Glucose metabolism

**Figure 5:**
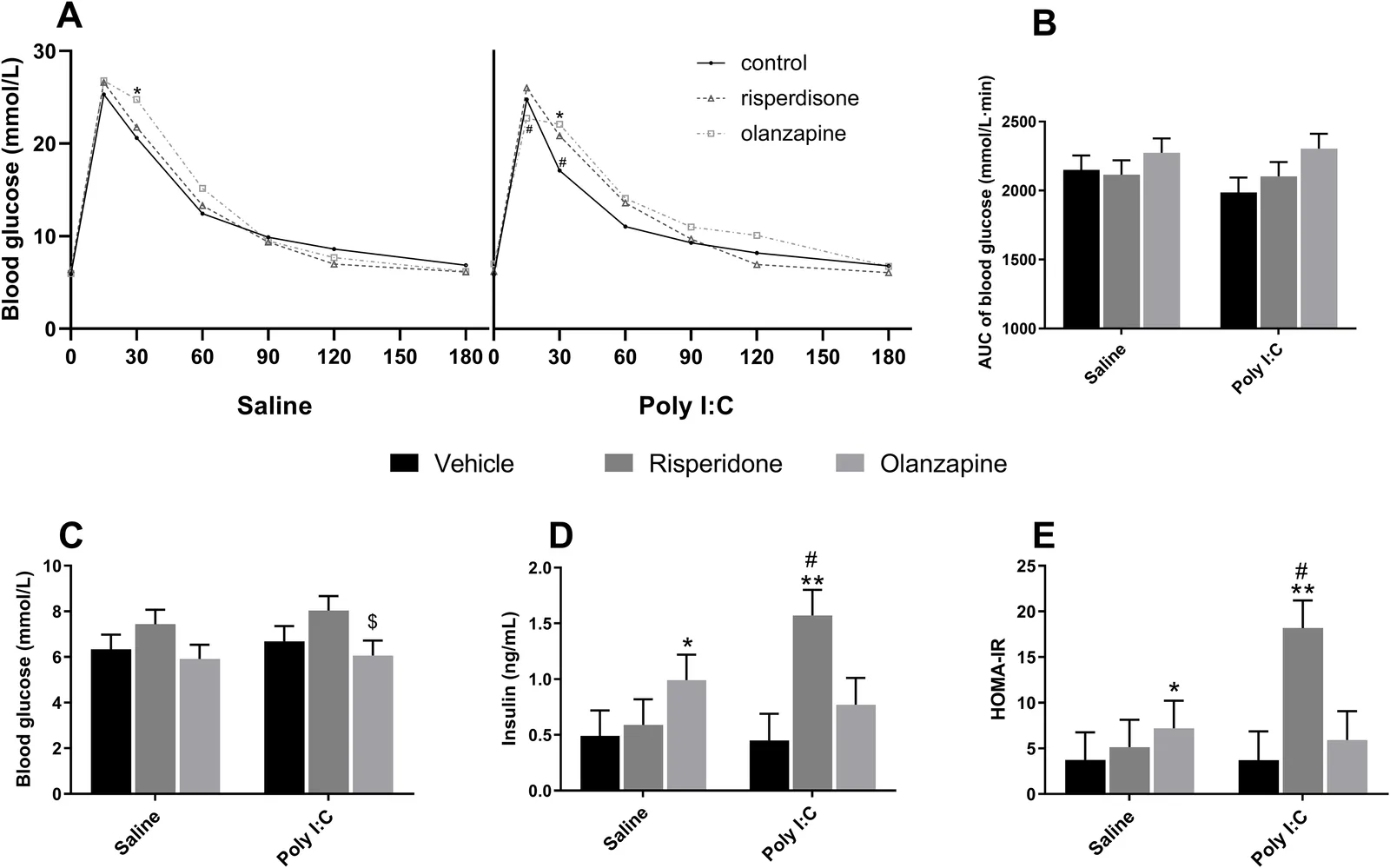
Effect of long-term SGA treatment and/or prenatal Poly I:C on glucose metabolism in female adult offspring. Panels show **(A)** blood glucose of IGTT, **(B)** AUC of IGTT, **(C)** fasting blood glucose, **(D)** insulin, and **(E)** HOMA-IR. Data are presented as EMMs ± SEM. For variables analyzed using natural log-transformation, values are presented as back-transformed geometric means, and SEMs were approximated using the delta method (or derived from back-transformed confidence intervals) to maintain visual consistency. Sample sizes: *n* = 12 per group, except for Poly I:C + OLZ and Poly I:C + Veh groups (both *n* = 11). ^*^p < 0.05, ^**^p < 0.01 *vs* Veh within the same prenatal treatment; ^$^*p* < 0.05 *vs* Poly I:C + RISP; ^#^*p* < 0.05 *vs* another prenatal treatment under the same drug. Poly I:C, Polyriboinosinic-Polyribocytidylic acid (5 mg/kg, at GD 15); AUC, area under the curve; HOMA-IR, Homeostatic Model Assessment for Insulin Resistance; Veh, vehicle; OLZ, olanzapine; RISP, risperidone; EMM, estimated marginal mean; SEM, Standard Error of the Mean.

For IGTT, there were significant main effects of time [*F*(6, 333.2) = 289.388, *p* < 0.001] and a significant interaction effect between time and drugs [*F*(12, 333.2) = 1.990, *p* = 0.025]. No significant three-way interaction (Poly I:C × Drug × Time) was observed (*p* > 0.05). OLZ treatment significantly increased glucose levels at the time point of 30 min in both Saline rats (*p* = 0.001 *vs* Saline + Veh) and Poly I:C rats (*p* = 0.034 *vs* Poly I:C + Veh). Meanwhile, at the time point of 30 min, the glucose levels were distinctly decreased in Poly I:C + Veh group compared to Saline + Veh group (*p* = 0.037). Moreover, at the time point of 15 min, the glucose level was significantly decreased in OLZ-treated Poly I:C compared to OLZ-treated Saline rats (*p* = 0.017; **Figure 5A**). However, no significant main effects of Poly I:C and SGAs, or their interaction on AUC of IGTT (all *p* > 0.05; **Figure 5B**). For the fasting glucose levels, there was a significant main effect of SGA administration [*F*(2, 52.5) = 5.230, *p* = 0.008], with OLZ treatment slightly decreasing glucose levels in Poly I:C rats compared with the RISP group (*p* = 0.047; **Figure 5C**). For the insulin concentration, there were significant main effects of SGA administration [*F*(2, 53.3)= 5.880, *p* = 0.005]. *Post-hoc* tests showed that RISP treatment significantly increased the insulin concentration in Poly I:C rats than Saline rats receiving the same treatment (*p* = 0.016), as well as compared with Poly I:C rats with vehicle treatment(*p* = 0.005; **Figure 5D**). In the Saline group, rats with OLZ treatment showed higher insulin levels than vehicle-treated controls (*p* = 0.040). For HOMA-IR value, a significant main effect of SGAs [*F*(2, 52.7) = 7.230, *p* = 0.002] and a significant interaction effect between Poly I:C and SGAs [*F*(2, 52.7) = 3.580, *p* = 0.035] were observed. *Post-hoc* comparisons revealed that HOMA-IR values in Poly I:C rats with RISP treatment were significantly increased than those in Saline rats under the same treatment (*p* = 0.010), as well as vehicle-treated Poly I:C rats (*p* = 0.001; **Figure 5E**). In Saline rats, OLZ treatment also increased HOMA-IR compared to the vehicle group (*p* = 0.038). These results suggested that adult female Poly I:C offspring showed differential metabolic susceptibility to specific SGAs. While OLZ impaired glucose tolerance independently of prenatal exposure, RISP treatment was specifically associated with insulin resistance within the Poly I:C model, reflecting a distinct interaction between neurodevelopmental vulnerability and drug-induced metabolic dysregulation.

### 3.3 Effect of SGAs and/or Poly I:C on growth and feeding parameters

**Figure 6:**
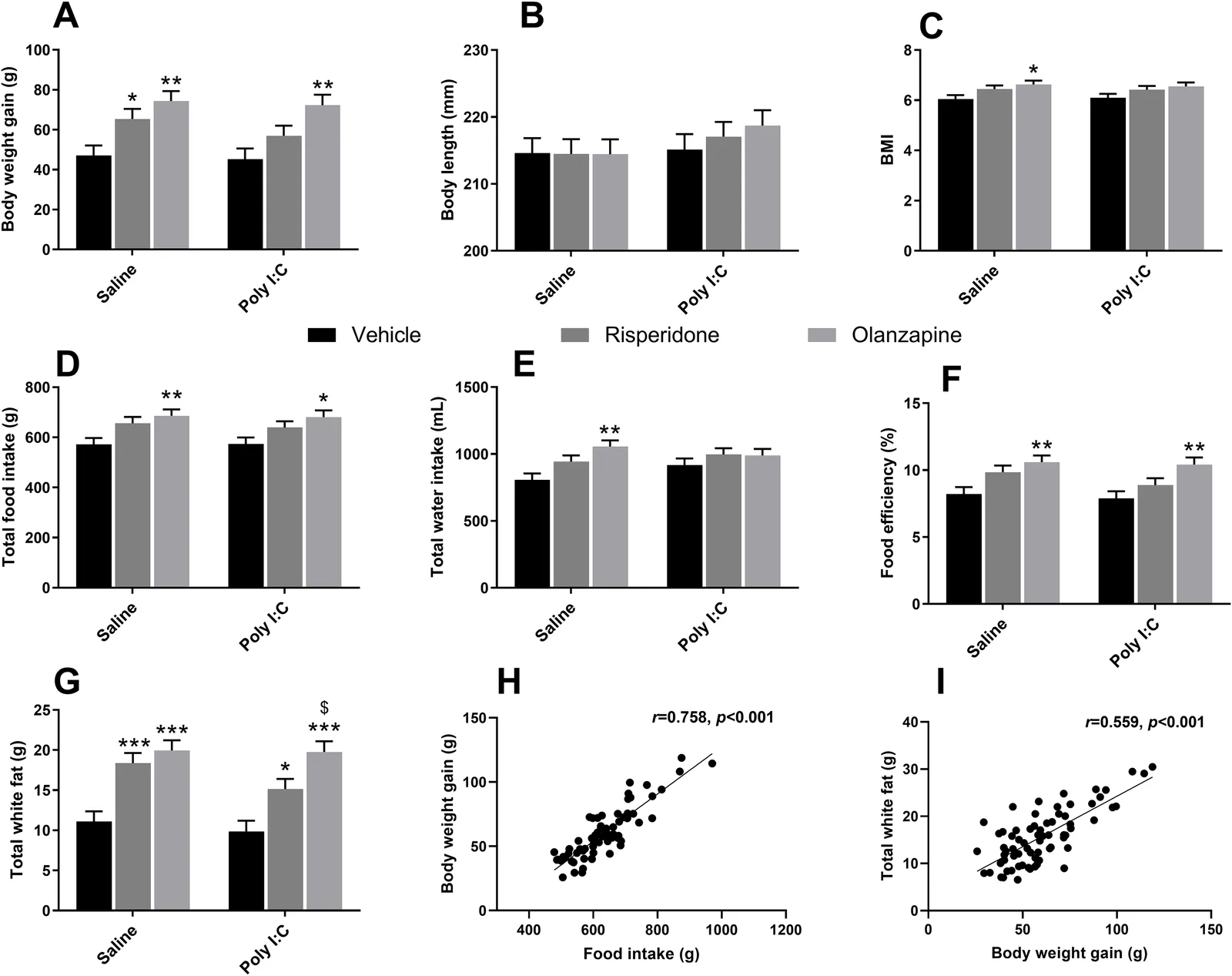
Effect of long-term SGA treatment and/or prenatal Poly I:C exposure on growth and feeding parameters. Panels show **(A)** body weight gain, **(B)** body length, **(C)** BMI, **(D)** total food intake, **(E)** total water intake, **(F)** feeding efficiency, **(G)** weight of total white fat (including perirenal, peri-ovary, mesentery and inguinal), **(H)** correlation between body weight gain and food intake, and **(I)** correlation between body weight gain and total white fat pad. Data are presented as EMMs ± SEM. For variables analyzed using natural log-transformation, values are presented as back-transformed geometric means, and SEMs were approximated using the delta method (or derived from back-transformed confidence intervals) to maintain visual consistency. Sample sizes: *n* = 12 per group, except for Poly I:C + OLZ and Poly I:C + Veh groups (both *n* = 11). ^*^p < 0.05, ^**^p < 0.01, ^***^p < 0.001 *vs* the corresponding Vehicle group within the same prenatal condition; ^$^*p* < 0.05 *vs* Poly I:C + RISP. Poly I:C, Polyriboinosinic-Polyribocytidylic acid (5 mg/kg, at GD 15); BMI, body mass index; Veh, vehicle; OLZ, olanzapine; RISP, risperidone; EMM, estimated marginal mean; SEM, Standard Error of the Mean.

For body weight gain, only a significant main effect of SGAs [*F*(2, 64.0) = 13.820, *p* < 0.001] was observed. Further *post-hoc* tests revealed that chronic SGA administration markedly increased body weight gain. Specifically, OLZ-treated rats displayed greater weight gain compared to their respective controls in both Saline (*p* = 0.001) and Poly I:C (*p* = 0.002) group. Similarly, RISP administration significantly elevated body weight gain in the Saline rats (*p* = 0.038 *vs* Saline + Veh) (**Figure 6A**). For body length, although no significant effects of both Poly I:C and SGAs, or their interaction was found in this study (**Figure 6B**), there was a significant main effect of SGAs on BMI [*F*(2, 64.0) = 5.720, *p* = 0.005], as BMI value was significantly higher in Saline rats following OLZ medication (*p* = 0.027 *vs* Saline + Veh; **Figure 6C**).

For food intake, there was a significant main effect of SGAs treatment [*F*(2, 64.0) = 9.640, *p* < 0.001], as evidenced by a significant increase following OLZ treatment. To detail the findings, Saline rats treated with OLZ consumed more food compared to both Saline + Veh (*p* = 0.006), and Poly I:C rats with the same treatment showed substantially increased food intake than Poly I:C + Veh groups (*p* = 0.016; **Figure 6D**). Likewise, a significant main effect of SGAs treatment [*F*(2, 64.0) = 5.740, *p* = 0.005] on water intake was observed in this study. There was a significant increase in water intake of Saline rats with the administration of OLZ (*p* = 0.001 *vs* Saline + Veh; **Figure 6E**). Consistently, there was a significant main effect of SGAs on feeding efficiency (body weight gain/food intake) during drug treatment [*F*(2, 64.0) = 11.120, *p* < 0.001], as OLZ treatment increased feed efficiency in both Saline group (*p* = 0.005 *vs* Saline + Veh) and Poly I:C group (*p* = 0.004 *vs* Poly I:C + Veh; **Figure 6F**).

For total white fat pad, there was a significant main effect of SGAs [*F*(2, 53.5) = 27.610, *p* < 0.001]. More precisely, OLZ administration inducing a prominent increase of white fat mass in both Saline and Poly I:C rats compared to their respective vehicle groups (both *p* < 0.001). Consistent with these observations, RISP treatment also significantly elevated fat weight against vehicle controls in both Saline (*p* < 0.001) and Poly I:C group (*p* = 0.016; **Figure 6G**). Besides, Poly I:C rats treated with OLZ showed a significantly greater total white fat mass than RISP-treated rats (*p* = 0.041). There was a significant positive correlation between food intake and body weight gain (*r* = 0.758, *p* < 0.001) (**Figure 6H**), as well as between body weight gain and total white fat pad mass (*r* = 0.559, *p* < 0.001) (**Figure 6I**), indicating that fat accumulation was the primary driver of the observed increase in body weight gain. Importantly, there was no significant main Poly I:C effect on body weight gain, body length, BMI, food and water intake, feed efficiency or fat mass (all *p* > 0.05).

These results suggested that long-term SGAs treatment, including OLZ and RISP, but not prenatal Poly I:C exposure, contributed to weight gain and obesity due to the increase of food intake in female adult rats.

### 3.4 Effect of SGAs and/or Poly I:C on voluntary wheel running activity

**Figure 7:**
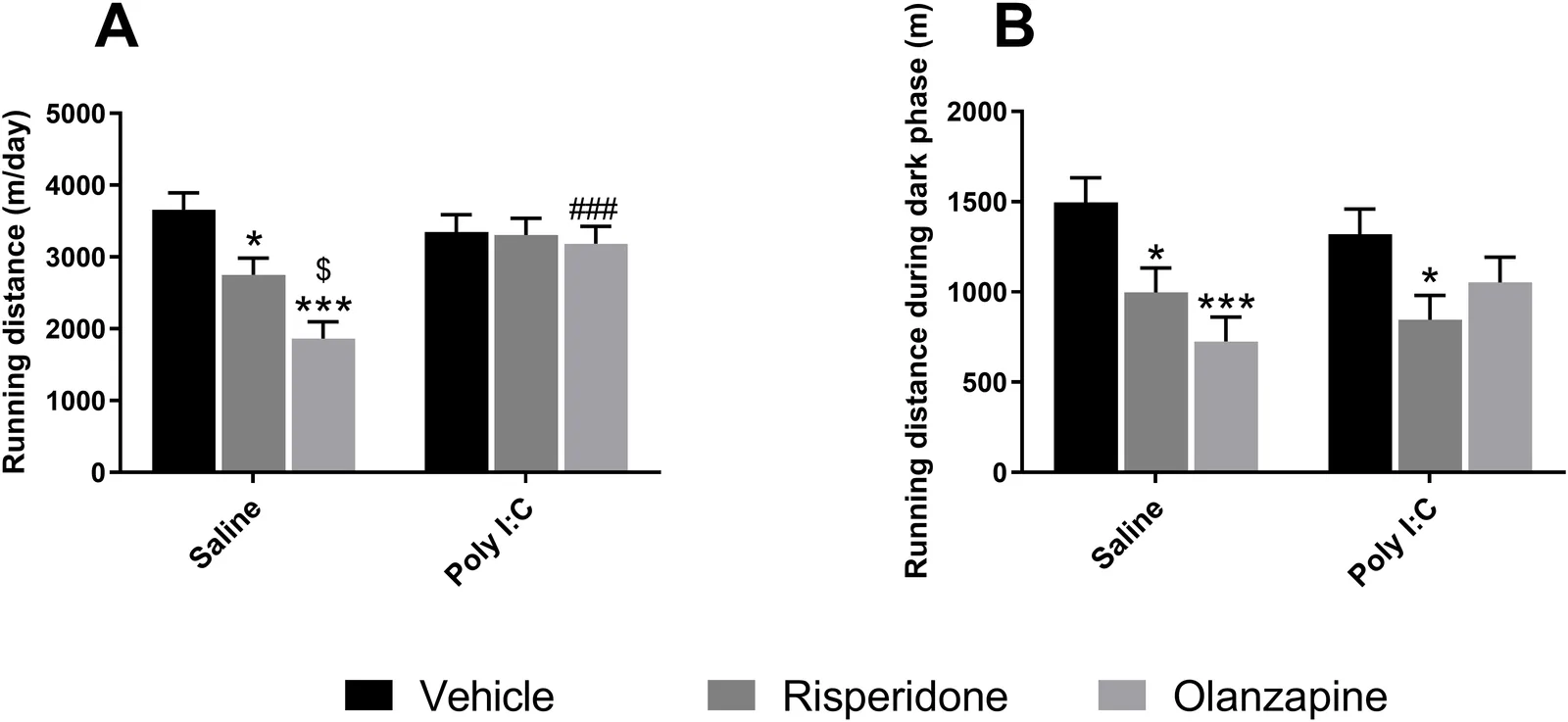
Voluntary wheel running test over 24 hours in female adult offspring. Panels show **(A)** total running distance over 24 hours and **(B)** total running distance during the dark phase. Data are presented as mean ± SEM. Sample sizes: *n* = 12 per group, except for Poly I:C + OLZ and Poly I:C + Veh groups (both *n* = 11). ^*^p < 0.05, ^***^p < 0.001 *vs* the corresponding Vehicle group within the same prenatal condition; ^$^*p* < 0.05 *vs* Saline + RISP; ^###^*p* < 0.001 vs Saline + OLZ. Poly I:C, Polyriboinosinic-Polyribocytidylic acid (5 mg/kg, at GD 15); Veh, vehicle; OLZ, olanzapine; RISP, risperidone.

In the twenty-four-hours voluntary wheel running test, there were significant main effects of both SGAs [*F*(2, 64.0) = 8.430, *p* = 0.001] and prenatal Poly I:C factors [*F*(1, 64.0) = 7.250, *p* = 0.009], as well as a significant interaction effect between Poly I:C and SGAs [*F*(2, 64.0) = 5.880, *p* = 0.005] on the total running distance. In Saline rats, OLZ treatment significantly reduced total running distance (*p* < 0.001 *vs* Saline + Veh; *p* = 0.027 *vs* Saline + RISP). Similarly, RISP administration also distinctly decreased the total running distance in saline group (*p* = 0.023 *vs* Saline + Veh; **Figure 7A**). Furthermore, under the same treatment of OLZ, the total running distance of Saline rats was substantially shorter than Poly I:C rats (*p* < 0.001). As rats are known to be active mostly during the dark phase, the total running distance during dark phase was analyzed separately in this study, whereas only a significant main effect of SGAs treatment [*F*(2, 53.2) = 11.070, *p* < 0.001] was observed. Dark-phase running distance was significantly decreased in RISP-treated rats in both Saline (*p* = 0.016 vs Saline + Veh) and Poly I:C group (*p* = 0.028 *vs* Poly I:C + Veh; **Figure 7B**). Besides, Dark-phase running distance was significantly decreased in OLZ-treated Saline rats (*p* < 0.001 vs Saline + Veh). These results suggested that beyond the SGAs effect, there were also a significant main effect of Poly I:C on locomotor activity and patterns.

### 3.5 Effect of SGAs and/or Poly I:C on endocrine hormones

**Figure 8:**
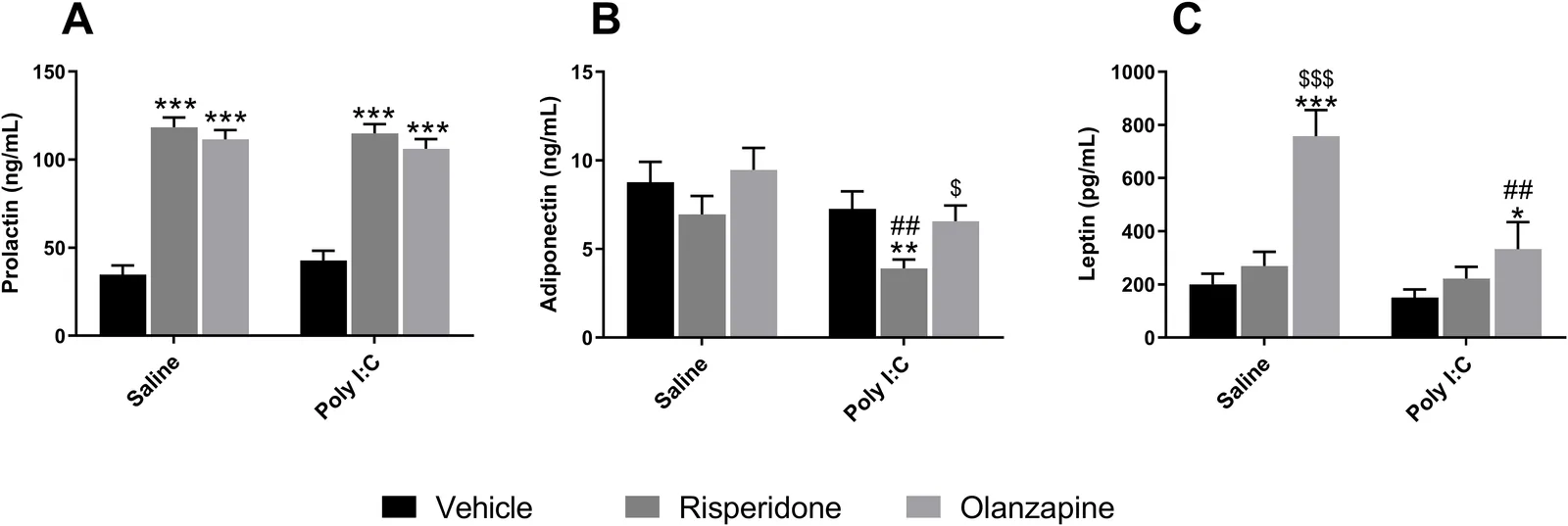
The Effect of long-term SGA treatment and/or prenatal Poly I:C exposure on endocrine hormones in female adult offspring. Panels show serum levels of **(A)** prolactin, **(B)** adiponectin, and **(C)** leptin. Data are presented as EMMs ± SEM. For variables analyzed using natural log-transformation, values are presented as back-transformed geometric means, and SEMs were approximated using the delta method (or derived from back-transformed confidence intervals) to maintain visual consistency. Sample sizes: *n* = 12 per group, except for Poly I:C + OLZ and Poly I:C + Veh groups (both *n* = 11). ^*^p < 0.05, ^**^p < 0.01, ^***^p < 0.001 *vs* the corresponding Vehicle group within the same prenatal condition; ^$^*p* < 0.05, ^$$$^*p* < 0.001 *vs* another treatment within the same prenatal condition; ^##^*p* < 0.01 *vs* another prenatal treatment under the same drug. Poly I:C, Polyriboinosinic-Polyribocytidylic acid (5 mg/kg, at GD 15); Veh, vehicle; OLZ, olanzapine; RISP, risperidone; EMM, estimated marginal mean; SEM, Standard Error of the Mean.

For prolactin levels, only a significant main effect of SGA factor was identified [*F*(2, 63.0) = 128.330, *p* < 0.001]. *Post-hoc* analysis showed that chronic administration of SGAs (both OLZ and RISP) markedly elevated prolactin concentrations in both Saline and Poly I:C rats, compared to their respective vehicle-treated controls (all *p* < 0.001; **Figure 8A**). For adiponectin levels, there were significant main effects of both prenatal Poly I:C exposure [*F*(1, 12.4) = 8.920, *p* = 0.011] and drug treatment [*F*(2, 50.9) = 8.250, *p* = 0.001], but no significant interaction effects between Poly I:C and drug (*p* > 0.05). Specifically, adiponectin concentration was significantly decreased in Poly I:C rats treated with RISP (*p* = 0.002 *vs* Poly I:C + Veh; *p* = 0.011 *vs* Poly I:C + OLZ). Notably, under the same RISP treatment, the Poly I:C group showed a significant reduction in adiponectin levels when compared to the Saline group (*p* = 0.003; **Figure 8B**). For leptin concentration, significant main effects were observed for both SGA administration [*F*(2, 51.7) = 17.880, *p* < 0.001] and prenatal Poly I:C exposure [*F*(1, 13.3) = 5.030, *p* = 0.043], while no significant interaction effects of these two factor (*p* > 0.05). OLZ treatment induced a pronounced increase in leptin concentrations with the Saline rats (*p* < 0.001 *vs* Saline + Veh, *p* < 0.001 *vs* Saline + RISP) and the Poly I:C rats (*p* = 0.011 *vs* Poly I:C + Veh). Furthermore, leptin levels in OLZ-treated Poly I:C rats were significantly lower than those detected in the Saline + OLZ group (*p* = 0.006; **Figure 8C**). These data revealed that beyond the effects of SGA treatment, prenatal Poly I:C exposure independently contributed to changes in hormones relevant to metabolic regulation, especially adiponectin and leptin. Moreover, prenatal Poly I:C exposure may alter the metabolic outcomes of SGA medication, as reflected by these endocrine changes. **Supplementary Table 10** showed linear mixed model results for PD70 metabolic outcomes. Notably, sensitivity analyses for all metabolic and behavioral parameters assessed during the drug treatment period—including lipid profiles, glucose regulation, energy intake, wheel-running activity, and endocrine measures—confirmed that the results were not driven by extreme values (**Supplementary Tables 11**, **12**).

## 4 Discussion

**Figure 9:**
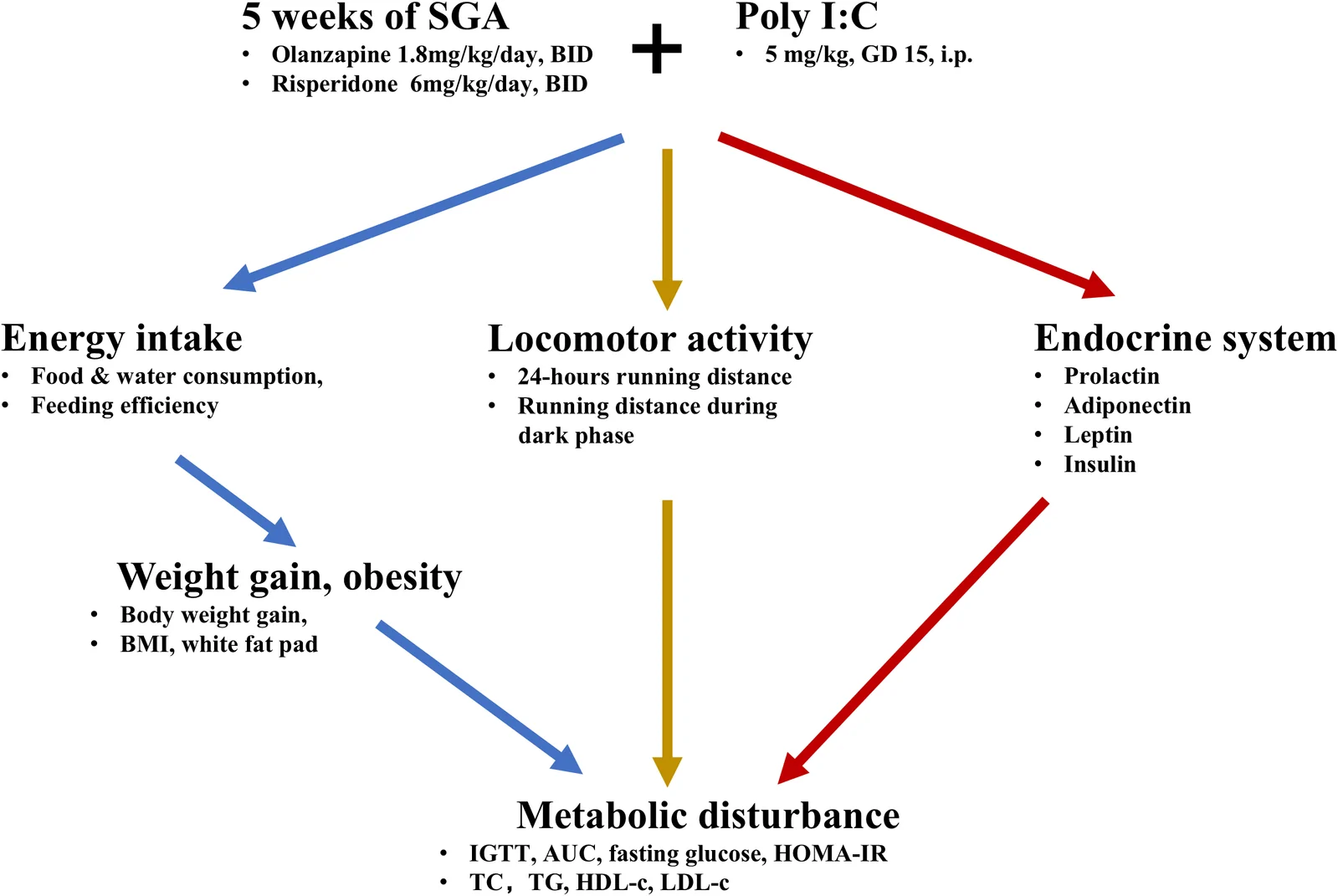
Interactive effects of prenatal Poly I:C exposure and long-term SGA treatment on the development of metabolic dysfunction. The interaction between prenatal Poly I:C exposure and SGAs drives metabolic dysfunction potentially through three main candidate pathways: (1) increasing energy intake and feeding efficiency (food/water consumption), which leads to obesity (body weight gain, BMI and total white fat pad); (2) reducing locomotor activity (total and dark phase running distance); and (3) disrupting endocrine regulation (prolactin, adiponectin, leptin and insulin). Together, these changes cause metabolic disturbance, resulting in abnormal lipid and glucose metabolism (TC, TG, HDL-c, LDL-c, glucose, IGTT, AUC and HOMA-IR). SGAs, second-generation antipsychotics; BID, twice daily; GD, gestation day; i.p., intraperitoneal; IGTT, intraperitoneal glucose tolerance test; AUC, area under the curve; HOMA-IR, homeostatic model assessment for insulin resistance; BMI, body mass index; TC, total cholesterol; TG, triglyceride; HDL-c, high-density lipoprotein cholesterol; LDL-c, low-density lipoprotein cholesterol.

In this study, we observed metabolic disturbances in first-episode drug-naïve children with ADHD and ASD, accompanied by elevated blood glucose and reduced ApoA-I levels in both NDD groups, with subgroup-specific alterations observed in TG and ApoB levels. The Poly I:C rat model partly reflected these findings at baseline (PD60), as an biologically relevant MIA framework for broad NDD-related vulnerability, showing significant reductions in TC and TG, as well as a sex-dependent decrease in HDL-c. Additionally, offspring displayed sex differences in glucose, TC and LDL-c levels at PD60. Furthermore, in the long-term SGA dataset—an experimental pharmacologic challenge in adult rats rather than treated pediatric cohorts—prenatal Poly I:C exposure was associated with changes in LDL-c and glucose, whereas long-term OLZ or RISP treatment was associated with a wider range of metabolic alterations in adult female offspring, including TC, HDL-c, insulin, HOMA-IR values (with a Poly I:C × SGA interaction effect), IGTT (with a Time × SGA interaction effect), and growth-related parameters, but no clear main effects for body length or TG. Moreover, locomotor patterns (24-h wheel running) and the adipokines (adiponectin and leptin) were influenced by both Poly I:C exposure and SGAs, with evidence of an interaction for locomotor activity, while insulin and prolactin changes were mainly linked to SGA treatment. Collectively, these results might suggest sex-related vulnerability in glucose and lipid regulation in NDDs and support the possibility that long-term SGA treatment compounds metabolic risk through coordinated changes in energy balance/weight gain, altered locomotor patterns, and altered endocrine profiles (**Figure 9**).

Consistent with most clinical evidence from ASD or ADHD (51, 52), and observations from animal studies (53), differences in lipid profiles and blood glucose levels were observed in the Poly I:C model and patients with NDDs prior to any pharmacological exposure (**Figures 2** and **3**). Multivariable models adjusted for age, BMI, and fasting time confirmed these alterations remained significant across NDD groups. These findings may indicate baseline metabolic differences associated with NDDs. Crucially, some metabolic parameters showed sex-divergent patterns, with sex differences evident for some measures. Accumulating evidence suggests that males are more physiologically vulnerable to metabolic disturbances under prenatal stress, while females may be partly protected by estrogen, which exerts anti-inflammatory and insulin-sensitizing effects (54, 55). Although results for lipid profiles are not fully consistent with existing literature, which commonly reports increased levels of TC and LDL-c among individuals diagnosed with NDDs (53, 56), we speculate that this discrepancy may be related to the specific developmental stage – adolescence, during which their metabolic baseline may be variable. These differences likely reflect developmental variations and provide evidence of a fundamental metabolic homeostatic disruption. Young males may have experienced a distinct acute-phase endocrine-metabolic stress response due to the prenatal immunological challenge potentially influencing the hypothalamic-pituitary-adrenal/gonadal axis during this vulnerable window period (57). Sex differences are essential factors in the metabolic investigation of the rodent MIA models for neurodevelopmental and neuropsychiatric disorders (58, 59); however, most animal studies do not specify the sex used while others are conducted in male offspring solely (60–62). Clinical and preclinical studies have also revealed that metabolic disturbances, for example, obesity, diabetes and metabolic syndrome, are more prominent in females with SCZ and most neuropsychiatric disorders, particularly when using antipsychotic medication (63–66). Our previous work demonstrated that prenatal Poly I:C stimulation affected the behavior of female juvenile rats, and early exposure to antipsychotic drugs during the juvenile period had long-term effects on adult behavior (42–44). Combined with the relatively less consistent metabolic alterations observed in adolescent females in the current study, we hypothesize that latent susceptibility in females is likely to manifest as metabolic dysregulation during exposure to persistent neurodevelopmental challenges or additional stressors (e.g., pharmacological interventions) in adulthood. Taken together, this study suggested that Poly I:C-induced NDD rats displayed sex-related biochemical metabolic abnormalities, consistent with prior research (40, 67). Consistent with high-impact evidence emphasizing the clinical need for pediatric metabolic monitoring (9), these considerations support routine metabolic assessments in NDD youth even before starting antipsychotic treatment. Given the heightened comorbidity of emotional symptoms (e.g., anxiety, depression) and pronounced susceptibility to SGA-induced adverse metabolic effects (e.g., weight gain, insulin resistance) observed in female patients with SCZ (45), along with the fact that antipsychotic drugs are widely used in the treatment of adult mental disorders (68), assessing SGA-induced metabolic alterations in adult female offspring is critical to minimize potential confounding effects caused by sex differences during adolescence.

Although several clinical studies observed the metabolic alterations existing in drug-naïve or drug-free patients with SCZ (56, 69), animal studies on evaluating the neurodevelopmental effect on metabolic abnormalities were still limited and their results were inconsistent. Specifically, a study from Singh R et al. did not find an effect of disease itself on both serum glucose and lipid profile in an MK-801-induced mouse model of SCZ (16). While consistent with the observation in female SD rats (15), our study found a significant effect of Poly I:C on LDL-c levels in adult female offspring (**Figure 4D**). Consistent with most clinical and preclinical studies (53, 70), there was a significant drug effect on lipids, such as TC and HDL-c, in our research (**Figures 4A, C**). Moreover, our study suggested that SGAs induced a higher level of TC, HDL-c, LDL-c as well as insulin and HOMA-IR, supporting the evidence that SGAs contribute to metabolic alterations (71). We did not find the significant interaction effect between Poly I:C and drugs on lipid metabolism, suggesting that the effects of prenatal challenge and SGAs medication on dyslipidemia may be a simple superimposition rather than a synergistic effect. Similar with the observation in mice (72), in this study we observed a significant effect of prenatal Poly I:C exposure on fasting glucose levels (**Figure 5C**). Importantly, although SGA × Time interactions were observed in IGTT, the glucose levels may suggest a background-dependent response pattern. Specifically, at 30 minutes, while OLZ treatment significantly increased glucose levels compared to the vehicle in both Saline and Poly I:C groups, a lower glucose level was evident in the Poly I:C vehicle group relative to the saline vehicle group (**Figure 5A**). Additionally, at 15 minutes, the glucose level was significantly lower in the Poly I:C compared to the Saline rats under OLZ challenge. The significant main effects of SGAs on insulin and HOMA-IR confirm their well-known diabetogenic risk (**Figures 5D, E**) (73). Notably, a significant interaction effect between Poly I:C and SGAs on HOMA-IR were observed in our study, indicating that prenatal immune activation and medication challenge act synergistically to drive insulin resistance (IR) far more severely than the superimposition of their independent effects (56). Taken together, our results from animal model provided evidence that there may be a latent metabolic vulnerability associated with NDDs during SGAs medication (10, 69).

It was reported that rapid weight gain was observed in antipsychotics- naïve psychotic patients in the first few weeks and continues during the following months (22). Synthesizing evidence from self-report questionnaires and food surveys suggested that patients with NDDs exhibit increased appetite and craving for fatty food, as well as increased caloric intake and snacking (23). However, in this study, there was a significant antipsychotic effect, but not Poly I:C effect, on both weight gain and energy intake in adult female SD offspring (**Figure 6**). Consistent with the popular point in the role of increasing energy intake induced-AO on metabolic abnormalities (17, 18), our results show that SGA-induced metabolic dysregulation was associated with the significant increase in both body weight gain, BMI value and white fat accumulation (**Figures 6A, C, G**). Unlike clinical cohorts where dietary factors often confound results, this controlled animal model demonstrates that SGA-driven weight gain occurs independently of the MIA condition. Evidence reported that chronic schizophrenia was associated with higher levels of visceral fat, which is further exacerbated by antipsychotic medication (74, 75). This aligned with our observations that SGA treatment increased total white fat a in a manner that closely tracked with both stimulated appetite (food intake) and enhanced food efficiency (body weight gain) (**Figures 6H, I**). These results were consistent with the other animal studies (15, 76), and suggested that the observed weight/fat increases were primarily linked to SGA exposure rather than prenatal Poly I:C exposure.

As is well known, reduced locomotor activity can lower energy expenditure and may contribute to weight gain and metabolic disturbance (77, 78). Exercise is an effective non-pharmacological lifestyle intervention to improve metabolic parameters including hyperglycaemia and insulin insensitivity associated with type 2 diabetes mellitus, dyslipidemia (79, 80). In this study, we evaluated the disease effect on animal’s locomotor activity and style using a twenty-four-hour of running wheel test which including one light phase and one dark phase, since most studies on SGAs side effect suggest the inactive state during the light phase can cause to ‘floor effect’ that prevent the evaluation of the effect on locomotor activity (81). Overall, we observed significant main effects of SGAs, Poly I:C and a significant SGA × Poly I:C interaction on 24-h running distance (**Figure 7A**). OLZ and RISP reduces total running distance in saline animals, but Poly I:C rats showed longer total running distances relative to the Saline + OLZ group. This pattern reflects an altered locomotor style and impaired circadian activity rhythms rather than a simple uniform reduction in activity (82), and may also relate to hyperactivity-like phenotypes relevant to ADHD, which could interact with energy balance differently than hypoactivity (83). Importantly, SGAs affected 24-h wheel running with reduced dark-phase running distance consistent with prior rodent studies (**Figure 7B**) (84, 85). Specifically, dark-phase running was significantly decreased in OLZ/RISP-treated Saline rats, but in the Poly I:C rats, this reduction was only significant under risperidone treatment. Longstanding behavioral abnormalities, including increased locomotor responsiveness to stress, novel environment, and amphetamine have been observed after puberty in rodents following Poly I:C-induced prenatal immune activation (43, 86). It may because changes in autonomic physiology, behavior, and mood were the critical characteristic of psychotic disease (87), and universal locomotor activity change reflected these characteristics was observed in animal or human being suffering from this disease (24).

Meanwhile, as for the endocrinal hormones, an increase in circulating prolactin levels has frequently been shown in patients with antipsychotic treatment (88). Nevertheless, schizophrenia patients without previous treatment also have been reported to present hyperprolactinemia (28). However, in our study, the significantly higher prolactin concentration was resulted from OLZ or RISP treatment rather than the prenatal Poly I:C challenge (**Figure 8A**). PRL affects metabolic homeostasis by regulating key enzymes and transport proteins involved in glucose and lipid metabolism (89). Additionally, PRL stimulates the release of insulin from pancreatic β cells, which may account for the development of insulin resistance in many individuals with elevated prolactin levels receiving these medication therapies (89). Adiponectin, an adipocyte-specific factor, plays a critical role iin increasing insulin sensitivity, with decreased serum concentration associated with insulin resistance, hence involves in metabolic syndrome (29). Consistently, we observed that increased insulin resistance was accompanied by significantly lower adiponectin levels, particularly in Poly I:C rats treated with OLZ (**Figure 8B**). This observation may largely be attributable to the adipoinsular axis that acts as an inhibitory feedback for insulin secretion (90). In addition, in line with several observations of lower level of adiponectin and aberrant insulin resistance in drug-free psychotic patients such as schizophrenia/depression (14, 30), a significant effect of Poly I:C on adiponectin was found here. Similarly, leptin is another adipocyte-specific factor that control metabolic process (31). Modifications of leptin metabolism and its gene expression, as well as its receptor, have been reported among psychotic patients independent to psychotropic drugs or in absence of antipsychotics treatment (32). In this study, we also observed a significant effect of Poly I:C on leptin concentration (**Figure 8C**). High leptin levels are generally associated with obesity and increased fat pads, but also indicating leptin resistance (91), which is supported by our observations that animals with elevated leptin levels had increased body weight and food intake. Consistent with prior research reported that OLZ directly increased leptin gene expression (92), we found a significant SGAs effect on leptin reflecting the result of fat accumulation. Moreover, apart from SGAs effect, there was a significant interaction effect of Poly I:C and drugs on leptin. Existing literature has proved that hyperleptinemia contributes to SGAs-associated obesity and metabolic disorders (93). Our study demonstrated that individuals with prenatal immune activation showed higher leptin levels after antipsychotic medication, which strongly suggests a more severe state of leptin resistance, with the hypothalamus insensitive to the reduce feeding signal sent by leptin, thus exacerbating metabolic disorders (94, 95). It has been suggested that there may share a common biological basis and have a bidirectional association between metabolic syndrome and emotional behavior (96). Because it is increasingly clear that the activity of dopamine neurons is also associated with the common emotional change in psychiatric disorders (97, 98), and the activity of dopamine neurons is modulated not only by synaptic inputs but also by peripheral hormones, such as leptin and adiponectin (99–101). These may explain why in line with the observations of elevated depression-like behaviours and decreased expression of dopamine D2 receptor in the hippocampus in female adolescent offspring in our previous study (43), a significant effect of prenatal Poly I:C exposure on the relative lower leptin and adiponectin level were also observed in this study.

There are several limitations in our study. Firstly, our clinical data were limited to ASD and ADHD. While we adjusted for main covariates like age, BMI and time of blood draw in our updated analysis, information on adiposity proxies, pubertal status, diet, physical activity, socioeconomic factors and comorbidities common in NDDs was unavailable due to retrospective study. These factors could introduce bias, as differences in lifestyle or how healthcare-seeking behavior may influence metabolic results. Additionally, the small sample size of females in our NDD groups may have limited our ability to detect certain sex-specific differences. Thus, our findings reflect potential associations rather than certain features of the disease itself. Since the metabolic changes cannot be generalized to the broader NDD subtypes, future prospective research are needed to better confirm these metabolic risks across larger, more diverse groups while collecting a fuller range of developmental and lifestyle data. Secondly, we acknowledge the age gap between the children (3–13 years) and the rats (PD60). However, finding similar metabolic disturbances in both children and rats before any medication suggests that this vulnerability is not limit to a temporary developmental phase. Instead, it may represent a persistent feature of the NDD background that lasts from childhood into adulthood. This framework serves as a translational bridge between clinical baseline vulnerability and experimental pharmacologic challenge. Thirdly, although we recognize the sex differences of the rat schizophrenia model induced by Poly I:C exposure during pregnancy, we still explore the psychotic effect on metabolic disturbance during SGAs treatment only in female regarding that estrogen greatly influence the efficacy and tolerability of SGAs. Males should be included in subsequent studies to understand the gender differences during SGAs medication. Fourth, we collected samples 2 hours after the final dose because OLZ and RISP have short half-lives in rats. This timing allows us to capture peak steady-state drug levels and high D2 receptor occupancy, which are key drivers of metabolic changes. This steady-state peak approach is essential for assessing metabolic dysregulation under continuous pharmacological challenge, reflecting the clinical scenario of active treatment. However, we recognize that this 2-hour window may still capture acute drug responses for certain markers, especially glucose and prolactin. Nevertheless, the observed alterations in parameters that are not susceptible to acute fluctuations, such as lipid profiles and adipose tissue mass, provide internal evidence for the chronic adaptive nature of the observed metabolic dysfunction. While our previous work showed that lipid levels stay consistent even after a 15-hour washout, this may not be the case for all endocrine and glucose measures. Future studies should include a longer washout period, such as 24 hours, to better separate short-term drug effects from long-term metabolic adaptations and further delineate the reversibility of these effects. Finally, our findings on behavior and hormones are exploratory. The running wheel data reflect complex movement styles and circadian patterns rather than a simple reduction in activity. Additionally, changes in adiponectin or leptin should be seen as candidate pathways rather than confirmed causal links, as we did not perform mediation analysis to link these hormones directly to metabolic outcomes.

## 5 Conclusion

In summary, this study provides converging evidence across clinical baseline vulnerability and experimental pharmacologic challenge in an etiologically relevant model, suggesting that metabolic disturbances in NDDs may not be solely a consequence of pharmacological intervention. These findings support the possibility that an underlying metabolic predisposition interacts with SGA treatment to influence glucose and lipid profiles. By positioning these results within the broader context of pediatric adverse effects, our results provide a translational scaffold supporting the clinical importance of metabolic monitoring even before pharmacologic exposure. Furthermore, NDD-related locomotor styles and candidate endocrine profiles may synergistically contribute to metabolic risk during SGA treatment, highlighting the need to consider these biological factors in long-term clinical management.

## Data Availability

The original contributions presented in the study are included in the article/**Supplementary Material**. Further inquiries can be directed to the corresponding authors.
