# Correction: Locomotor and endocrine alterations link to metabolic dysfunction induced by pathopharmacological interaction between neurodevelopmental disorders and antipsychotics: evidence from clinical and animal study

**DOI:** 10.3389/fpsyt.2026.1979982

**Published:** 2026-09-04

**Authors:** Menglu Zeng, Xinyu Yang, Zhenju Cao, Huiyu Chen, Yanfang Lu, Chen Xu, Danlin Weng, Anying Shen, Fei Xue, Wei Lin, Jianan Shi, Shuangyan Yang, Aifang Zhang, Fuchun Zhong, Yueqing Su

**Affiliations:** 1 Fujian Maternity and Child Health Hospital, College of Clinical Medicine for Obstetrics & Gynecology and Pediatrics, Fujian Medical University, Fuzhou, China; 2 Fujian Maternity and Child Health Hospital, Fuzhou, China; 3 School of Public Health, Fujian Medical University, Fuzhou, China; 4 Fuzhou First General Hospital, Affiliated with Fujian Medical University, Fuzhou, China

**Keywords:** adiponectin, insulin, leptin, locomotor activity, metabolic disturbance, neurodevelopmental disorders, Poly I:C, second-generation antipsychotic

Affiliations 1 and 2 were originally listed in the wrong order.

The original affiliation 1 “Fujian Maternity and Child Health Hospital, Affiliated Hospital of Fujian Medical University, Fuzhou, China” has been corrected to read “Fujian Maternity and Child Health Hospital, Fuzhou, China” and renumbered as affiliation 2.

The original affiliation 2 “College of Clinical Medicine for Obstetrics & Gynaecology and Paediatrics, Fujian Medical University, Fuzhou, China” has been corrected to read “Fujian Maternity and Child Health Hospital, College of Clinical Medicine for Obstetrics & Gynecology and Pediatrics, Fujian Medical University, Fuzhou, China” and renumbered as affiliation 1.

Author “Xinyu Yang” was originally assigned to affiliations 1,3. This has been corrected to 2,3.

Author “Anying Shen” was originally assigned to affiliations 1,3. This has been corrected to 2,3.

The original version of this article has been updated.

