# Supplementary material for: Locomotor and endocrine alterations link to metabolic dysfunction induced by pathopharmacological interaction between neurodevelopmental disorders and antipsychotics: evidence from clinical and animal study
Source: Front Psychiatry. 2026 Mar 10;17:1764492. doi: 10.3389/fpsyt.2026.1764492 (PMC13008908; doi:10.3389/fpsyt.2026.1764492)
Supplement: Supplementary file 1 [file DataSheet1.pdf]

Supplementary Table 1. Summary of covariate-adjusted GLMs for clinical metabolic outcomes: main effects of group, sex, and their interactions.

Outcome	Variable	*F*(df)	*p*	*ηp²*
**GLU†**				
	Group	$F(2,416) = 58.038$	$p < 0.001$	0.218
	Sex	$F(1,416) = 0.002$	$p = 0.968$	0.000
	Group × Sex	$F(2,416) = 0.507$	$p = 0.603$	0.002
**TG†**				
	Group	$F(2,416) = 3.041$	$p = 0.049$	0.014
	Sex	$F(1,416) = 0.000$	$p = 0.983$	0.000
	Group × Sex	$F(2,416) = 2.812$	$p = 0.061$	0.013
**TC†**				
	Group	$F(2,416) = 1.201$	$p = 0.302$	0.006
	Sex	$F(1,416) = 1.365$	$p = 0.243$	0.003
	Group × Sex	$F(2,416) = 0.275$	$p = 0.760$	0.001
**HDL-c**				
	Group	$F(2,416) = 0.851$	$p = 0.428$	0.004
	Sex	$F(1,416) = 0.017$	$p = 0.897$	0.000
	Group × Sex	$F(2,416) = 1.442$	$p = 0.238$	0.007
**LDL-c**				
	Group	$F(2,416) = 2.849$	$p = 0.059$	0.014
	Sex	$F(1,416) = 1.348$	$p = 0.246$	0.003
	Group × Sex	$F(2,416) = 0.021$	$p = 0.979$	0.000
**ApoA-I**				
	Group	$F(2,416) = 14.357$	$p = 0.000$	0.065
	Sex	$F(1,416) = 2.471$	$p = 0.117$	0.006
	Group × Sex	$F(2,416) = 1.198$	$p = 0.303$	0.006
**ApoB**				
	Group	$F(2,416) = 5.210$	$p = 0.006$	0.024
	Sex	$F(1,416) = 1.411$	$p = 0.236$	0.003
	Group × Sex	$F(2,416) = 0.103$	$p = 0.902$	0.000

Note. This table reports Type III tests from covariate-adjusted General Linear Models (GLM).

a Model Specification: Fixed factors were Group, Sex, and Group × Sex; covariates were Age, BMI, and Time of blood draw (AM/PM).

b Transformation: Outcomes marked with † had skewed residuals and were analyzed after natural log (ln) transformation; therefore, the reported *F*(df), *p* values, and partial eta-squared (ηp^2) statistics are based on the ln scale.

c Significance: Bold values indicate statistical significance ($p < 0.05$).

† Outcome analyzed using ln transformation.

Supplementary Table 2. Model-based EMMs (95% CI) stratified by sex and diagnostic group.

Outcome		Control EMM (95%CI)	ADHD EMM (95%CI)	ASD EMM (95%CI)
**GLU**†				
	Male	4.58(4.52,4.65)	5.09(5.02,5.17)***	5.14(5.03,5.25)***
	Female	4.53(4.41,4.65)	5.16(4.98,5.35)***	5.13(4.90,5.37)***
**TC**†				
	Male	4.28(4.14,4.43)	4.36(4.20,4.52)	4.06(3.85,4.27)
	Female	4.50(4.22,4.79)#	4.38(4.02,4.78)$	4.24(3.80,4.74)
**TG**†				
	Male	0.86(0.82,0.91)	0.81(0.77,0.86)	0.89(0.83,0.97)
	Female	0.75(0.68,0.83)#	0.86(0.75,0.98)	0.97(0.81,1.15)*
**HDL-c**				
	Male	1.38(1.34,1.42)	1.42(1.38,1.47)	1.32(1.26,1.38)$
	Female	1.41(1.34,1.49)	1.34(1.24,1.44)	1.36(1.23,1.49)
**LDL-c**				
	Male	2.33(2.25,2.41)	2.21(2.12,2.29)	2.13(2.01,2.26)*
	Female	2.40(2.25,2.55)	2.30(2.10,2.50)	2.21(1.95,2.47)
**ApoA-I**				
	Male	1.42(1.38,1.46)	1.35(1.31,1.39)*$	1.22(1.16,1.27)***
	Female	1.40(1.33,1.47)	1.23(1.14,1.33)*#	1.21(1.09,1.33)*
**ApoB**				
	Male	0.71(0.69,0.73)	0.66(0.64,0.69)*	0.66(0.63,0.70)
	Female	0.74(0.70,0.78)	0.68(0.62,0.73)	0.68(0.61,0.76)

Note: This table presents estimated marginal means (EMMs) and 95% confidence intervals (CIs) derived from covariate-adjusted generalized linear models (GLMs) adjusted for age, BMI, and time of blood draw. Outcomes marked with † were analyzed after natural log (ln) transformation; values are back-transformed to the original scale and represent geometric means (95% CI). Non-transformed outcomes are reported on the original scale. Pairwise comparisons with Bonferroni adjustment are provided in Table S3.

Significance symbols (Bonferroni-adjusted):

Within each sex stratum: *, **, *** indicate differences *vs* the Control group (*$p < 0.05$, ** $p < 0.01$, *** $p < 0.001$).

Within each diagnostic group: # indicates Female *vs* Male ($p < 0.05$).

Within each sex stratum: $ indicates ASD *vs*. ADHD ($p < 0.05$).

† Outcome analyzed using ln transformation.

Supplementary Table 3. Bonferroni-adjusted pairwise comparisons of EMMs, stratified by sex and diagnostic group.

Outcome	Stratum (sex/group)	Contrast	MD	95%CI	*p*_adj
**GLU**†					
	Male	ASD *vs* Control	1.083	(1.088, 1.158)	$p < 0.001$
		ADHD *vs* Control	1.141	(1.083, 1.141)	$p < 0.001$
		ASD *vs* ADHD	1.009	(0.977, 1.043)	$p = 1.000$
	Female	ASD *vs* Control	1.134	(1.062, 1.210)	$p < 0.001$
		ADHD *vs* Control	1.140	(1.080, 1.203)	$p < 0.001$
		ASD *vs* ADHD	0.994	(0.926, 1.068)	$p = 1.000$
	ASD	Male *vs* Female	1.002	(0.953, 1.054)	$p = 0.937$
	ADHD	Male *vs* Female	0.987	(0.949, 1.026)	$p = 0.516$
	Control	Male *vs* Female	1.012	(0.982, 1.043)	$p = 0.420$
**TC**†					
	Male	ASD *vs* Control	0.947	(0.878, 1.022)	$p = 0.270$
		ADHD *vs* Control	1.019	(0.958, 1.083)	$p = 1.000$
		ASD *vs* ADHD	0.931	(0.861, 1.005)	$p = 0.078$
	Female	ASD *vs* Control	0.944	(0.806, 1.106)	$p = 1.000$
		ADHD *vs* Control	0.975	(0.856, 1.112)	$p = 1.000$
		ASD *vs* ADHD	0.968	(0.814, 1.150)	$p = 1.000$
	ASD	Male *vs* Female	0.956	(0.846, 1.079)	$p = 0.462$
	ADHD	Male *vs* Female	0.994	(0.905, 1.092)	$p = 0.900$
	Control	Male *vs* Female	0.952	(0.886, 1.023)	$p = 0.179$
**TG**†					
	Male	ASD *vs* Control	1.039	(0.922, 1.171)	$p = 1.000$
		ADHD *vs* Control	0.942	(0.856, 1.037)	$p = 0.410$
		ASD *vs* ADHD	1.103	(0.976, 1.246)	$p = 0.163$
	Female	ASD *vs* Control	1.283	(1.001, 1.642)	$p = 0.049$
		ADHD *vs* Control	1.133	(0.923, 1.391)	$p = 0.427$
		ASD *vs* ADHD	1.131	(0.863, 1.483)	$p = 0.822$
	ASD	Male *vs* Female	0.925	(0.765, 1.120)	$p = 0.424$
	ADHD	Male *vs* Female	0.949	(0.820, 1.100)	$p = 0.484$
	Control	Male *vs* Female	1.142	(1.020, 1.278)	$p = 0.021$
**HDL-c**					
	Male	ASD *vs* Control	-0.060	(-0.150, 0.029)	$p = 0.312$

Outcome	Stratum (sex/group)	Contrast	MD	95%CI	*p*_adj
		ADHD *vs* Control	0.040	(-0.032, 0.112)	$p = 0.547$
		ASD *vs* ADHD	-0.100	(-0.192, -0.009)	$p = 0.025$
	Female	ASD *vs* Control	-0.053	(-0.238, 0.132)	$p = 1.000$
		ADHD *vs* Control	-0.072	(-0.225, 0.081)	$p = 0.785$
		ASD *vs* ADHD	0.019	(-0.183, 0.221)	$p = 1.000$
	ASD	Male *vs* Female	-0.038	(-0.180, 0.104)	$p = 0.601$
	ADHD	Male *vs* Female	0.081	(-0.028, 0.191)	$p = 0.146$
	Control	Male *vs* Female	-0.030	(-0.114, 0.054)	$p = 0.479$
**LDL-c**					
	Male	ASD *vs* Control	-0.198	(-0.378, -0.018)	$p = 0.025$
		ADHD *vs* Control	-0.127	(-0.272, 0.018)	$p = 0.107$
		ASD *vs* ADHD	-0.071	(-0.255, 0.113)	$p = 1.000$
	Female	ASD *vs* Control	-0.186	(-0.560, 0.187)	$p = 0.693$
		ADHD *vs* Control	-0.098	(-0.407, 0.211)	$p = 1.000$
		ASD *vs* ADHD	-0.088	(-0.496, 0.319)	$p = 1.000$
	ASD	Male *vs* Female	-0.077	(-0.364, 0.210)	$p = 0.599$
	ADHD	Male *vs* Female	-0.094	(-0.316, 0.127)	$p = 0.402$
	Control	Male *vs* Female	-0.065	(-0.235, 0.105)	$p = 0.451$
**ApoA-I**					
	Male	ASD *vs* Control	-0.200	(-0.283, -0.118)	$p < 0.001$
		ADHD *vs* Control	-0.073	(-0.139, -0.006)	$p = 0.027$
		ASD *vs* ADHD	-0.128	(-0.212, -0.044)	$p = 0.001$
	Female	ASD *vs* Control	-0.191	(-0.362, -0.019)	$p = 0.023$
		ADHD *vs* Control	-0.165	(-0.307, -0.024)	$p = 0.016$
		ASD *vs* ADHD	-0.025	(-0.212, 0.162)	$p = 1.000$
	ASD	Male *vs* Female	0.011	(-0.120, 0.143)	$p = 0.866$
	ADHD	Male *vs* Female	0.114	(0.013, 0.216)	$p = 0.028$
	Control	Male *vs* Female	0.021	(-0.057, 0.099)	$p = 0.591$
**ApoB**					
	Male	ASD *vs* Control	-0.048	(-0.098, 0.002)	$p = 0.063$
		ADHD *vs* Control	-0.049	(-0.090, -0.009)	$p = 0.011$
		ASD *vs* ADHD	0.001	(-0.051, 0.052)	$p = 1.000$
	Female	ASD *vs* Control	-0.061	(-0.165, 0.044)	$p = 0.488$

Outcome	Stratum (sex/group)	Contrast	MD	95%CI	*p*_adj
		ADHD *vs* Control	-0.066	(-0.152, 0.020)	$p = 0.194$
		ASD *vs* ADHD	0.006	(-0.108, 0.119)	$p = 1.000$
	ASD	Male *vs* Female	-0.020	(-0.100, 0.060)	$p = 0.622$
	ADHD	Male *vs* Female	-0.015	(-0.077, 0.047)	$p = 0.632$
	Control	Male *vs* Female	-0.032	(-0.080, 0.015)	$p = 0.180$

Note. This table reports post hoc contrasts derived from the GLM (Bonferroni adjustment applied; p_adj denotes two-sided multiplicity-adjusted p-values).

a Interpretation of Differences: For outcomes marked with † (analyzed after ln transformation), the Mean Difference (MD) and 95% CIs represent differences on the ln scale (ln(I) – ln(J) = ln(I/J)). If interpretation on the original scale is desired, applying the exponential function [exp(MD)] yields the ratio of group means (I/J) with its 95% CI.

b For non-transformed outcomes, MD represents simple mean differences on the original scale (I – J).

† Outcome analyzed using ln transformation.

Supplementary Table 4. Outlier counts (ROUT, Q=1%) per outcome and diagnosis-sex subgroup.

Outcome	Male + ASD (N = 68)	Male + ADHD (N = 129)	Male + Control (N = 146)	Female + ASD (N = 14)	Female + ADHD (N = 23)	Female + Control (N = 45)	Total outliers (n/N)
**GLU†**	3	-	1	-	-	-	**4/425**
**TC†**	-	1	5	-	-	1	**7/425**
**TG†**	-	-	1	-	-	-	**1/425**
**HDL-c**	-	-	-	-	-	-	**0/425**
**LDL-c**	-	1	-	-	-	-	**1/425**
**ApoA-I**	-	1	-	-	-	-	**1/425**
**ApoB**	-	1	-	-	-	-	**1/425**

Note: This table reports the number of outliers identified for sensitivity analyses, listed across six diagnosis-by-sex subgroups (Male-ASD, Male-ADHD, Male-Control, Female-ASD, Female-ADHD, Female-Control). Outliers were identified using the ROUT method in GraphPad Prism (Q = 1%). For outcomes analyzed using ln transformation, outlier identification was performed on the ln scale. Primary analyses retained all observations (no outlier removal); this table is provided to transparently describe the distribution of identified outliers by outcome and subgroup. "–" indicates that no outliers were identified. The total column reports total outliers/total N for each outcome. † Outcome analyzed using ln transformation.

Supplementary Table 5. Sensitivity analysis for outliers: comparison of GLM results before and after exclusion (ROUT, Q = 1%).

Outcome	Variable	Primary (all data) F(df), p, ηp^2	Sensitivity (outliers excluded) F(df), p, ηp^2	Change?
GLU†				
	Group	$F(2,416) = 58.038$ $p = 0.000$, $\eta p^2 = 0.218$	$F(2,412) = 70.258$ $p = 0.000$, $\eta p^2 = 0.254$	NO
	Sex	$F(1,416) = 0.002$ $p = 0.968$, $\eta p^2 = 0.000$	$F(1,412) = 0.003$ $p = 0.957$, $\eta p^2 = 0.000$	NO
	Group × Sex	$F(2,416) = 0.507$ $p = 0.603$, $\eta p^2 = 0.002$	$F(2,412) = 0.631$ $p = 0.532$, $\eta p^2 = 0.003$	NO
**TG†**				
	Group	$F(2,416) = 3.041$ $p = 0.049$, $\eta p^2 = 0.014$	$F(2,415) = 3.150$ $p = 0.044$, $\eta p^2 = 0.015$	NO
	Sex	$F(1,416) = 0.000$ $p = 0.983$, $\eta p^2 = 0.000$	$F(1,415) = 0.003$ $p = 0.957$, $\eta p^2 = 0.000$	NO
	Group × Sex	$F(2,416) = 2.812$ $p = 0.061$, $\eta p^2 = 0.013$	$F(2,415) = 2.551$ $p = 0.079$, $\eta p^2 = 0.012$	NO
**TC†**				
	Group	$F(2,416) = 1.201$ $p = 0.302$, $\eta p^2 = 0.006$	$F(2,409) = 1.033$ $p = 0.357$, $\eta p^2 = 0.005$	NO
	Sex	$F(1,416) = 1.365$ $p = 0.243$, $\eta p^2 = 0.003$	$F(1,409) = 2.370$ $p = 0.124$, $\eta p^2 = 0.006$	NO
	Group × Sex	$F(2,416) = 0.275$ $p = 0.760$, $\eta p^2 = 0.001$	$F(2,409) = 0.950$ $p = 0.387$, $\eta p^2 = 0.005$	NO
**LDL-c**				
	**Group**	**$F(2,416) = 2.849$ $p = 0.059$, $\eta p^2 = 0.014$**	**$F(2,415) = 3.077$ $p = 0.047$, $\eta p^2 = 0.015$**	**YES**
	Sex	$F(1,416) = 1.348$ $p = 0.246$, $\eta p^2 = 0.003$	$F(1,415) = 1.539$ $p = 0.215$, $\eta p^2 = 0.004$	NO
	Group × Sex	$F(2,416) = 0.021$ $p = 0.979$, $\eta p^2 = 0.000$	$F(2,415) = 0.030$ $p = 0.970$, $\eta p^2 = 0.000$	NO
**ApoA-I**				
	Group	$F(2,416) = 14.357$ $p = 0.000$, $\eta p^2 = 0.065$	$F(2,415) = 15.203$ $p = 0.000$, $\eta p^2 = 0.068$	NO
	Sex	$F(1,416) = 2.471$ $p = 0.117$, $\eta p^2 = 0.006$	$F(1,415) = 2.271$ $p = 0.133$, $\eta p^2 = 0.005$	NO
	Group × Sex	$F(2,416) = 1.198$ $p = 0.303$, $\eta p^2 = 0.006$	$F(2,415) = 1.084$ $p = 0.339$, $\eta p^2 = 0.005$	NO

**ApoB**				
	Group	$F(2,416) = 5.210$<br>$p = 0.006$, $\eta p^2 = 0.024$	$F(2,415) = 5.733$<br>$p = 0.003$, $\eta p^2 = 0.027$	NO
	Sex	$F(1,416) = 1.411$<br>$p = 0.236$, $\eta p^2 = 0.003$	$F(1,415) = 1.647$<br>$p = 0.200$, $\eta p^2 = 0.004$	NO
	Group × Sex	$F(2,416) = 0.103$<br>$p = 0.902$, $\eta p^2 = 0.000$	$F(2,415) = 0.093$<br>$p = 0.911$, $\eta p^2 = 0.000$	NO

Note: This table compares results from the primary analysis (all observations retained) with a sensitivity analysis excluding ROUT-identified outliers, using the same covariate-adjusted GLM. Fixed factors included Group, Sex, and Group × Sex, with covariates Age, BMI, and time of blood draw (AM/PM). Type III effects are reported as F(df), p-values, and partial eta-squared *(ηp²)*. For ln-transformed outcomes, F, *p*, and ηp² are based on models fitted on the ln scale. The "Change" column flags whether any substantive change occurred relative to the primary analysis (e.g., crossing the 0.05 threshold); "NO" indicates no substantive change. Primary inference is based on the primary (all-data) analyses; outlier exclusion was performed only as a sensitivity check to assess robustness. † Outcome analyzed using ln transformation.

Supplementary Table 6. Litter–offspring assignment and experimental group allocation.

Prenatal treatment	DAM_ID	RAT_ID	Sex	PD60_blood collection	PD70_Female Drug details
Saline	201				
		1	Female		Vehicle
		2	Female	√	Vehicle
		3	Female	√	Olanzapine
		4	Female		Olanzapine
		5	Female		Risperdisone
		441	Male	√	NA
		442	Male	√	NA
		443	Male	√	NA
Saline	202				
		6	Female	√	Vehicle
		7	Female	√	Olanzapine
		8	Female		Risperdisone
		9	Female		Risperdisone
		444	Male	√	NA
		445	Male	√	NA
		446	Male	√	NA
Saline	203				
		10	Female		Vehicle

Prenatal treatment	DAM_ID	RAT_ID	Sex	PD60_blood collection	PD70_Female Drug details
		11	Female		Vehicle
		12	Female	√	Vehicle
		13	Female		Olanzapine
		14	Female		Olanzapine
		15	Female		Risperdisone
		16	Female	√	Risperdisone
		447	Male	√	NA
		448	Male	√	NA
		449	Male	√	NA
Saline	204				
		17	Female		Vehicle
		18	Female		Olanzapine
		19	Female	√	Risperdisone
		450	Male	√	NA
		451	Male	√	NA
		452	Male	√	NA
Saline	205				
		20	Female		Vehicle
		21	Female		Vehicle
		22	Female		Olanzapine
		23	Female	√	Olanzapine
		24	Female	√	Risperdisone
		25	Female		Risperdisone
		453	Male	√	NA
		454	Male	√	NA
		455	Male	√	NA
Saline	206				
		26	Female	√	Vehicle
		27	Female		Olanzapine
		28	Female		Olanzapine
		29	Female	√	Risperdisone
		456	Male	√	NA
		457	Male	√	NA
		458	Male	√	NA
Saline	207				
		30	Female	√	Vehicle
		31	Female	√	Olanzapine
		32	Female		Risperdisone
		459	Male	√	NA
		460	Male	√	NA

Prenatal treatment	DAM_ID	RAT_ID	Sex	PD60_blood collection	PD70_Female Drug details
		461	Male	√	NA
Saline	208				
		33	Female		Vehicle
		34	Female	√	Olanzapine
		35	Female		Risperdisone
		36	Female	√	Risperdisone
		462	Male	√	NA
		463	Male	√	NA
Poly I:C	701				
		51	Female		Vehicle
		52	Female	√	Vehicle
		53	Female		Olanzapine
		54	Female	√	Olanzapine
		55	Female		Risperdisone
		421	Male	√	NA
		422	Male	√	NA
Poly I:C	702				
		56	Female		Vehicle
		57	Female	√	Olanzapine
		58	Female	√	Risperdisone
		59	Female		Risperdisone
		423	Male	√	NA
		424	Male	√	NA
Poly I:C	703				
		60	Female		Vehicle
		61	Female	√	Vehicle
		62	Female		Olanzapine
		63	Female		Olanzapine
		64	Female	√	Risperdisone
		65	Female		Risperdisone
		425	Male	√	NA
		426	Male	√	NA
		427	Male	√	NA
Poly I:C	704				
		66	Female		Vehicle
		67	Female		Vehicle
		68	Female	√	Olanzapine
		69	Female		Olanzapine
		70	Female	√	Risperdisone
		71	Female		Risperdisone

Prenatal treatment	DAM_ID	RAT_ID	Sex	PD60_blood collection	PD70_Female Drug details
		428	Male	√	NA
		429	Male	√	NA
Poly I:C	705				
		72	Female	√	Vehicle
		73	Female	√	Olanzapine
		74	Female		Risperidone
		430	Male	√	NA
		431	Male	√	NA
Poly I:C	706				
		75	Female	√	Vehicle
		76	Female		Olanzapine
		77	Female	√	Risperidone
		432	Male	√	NA
		433	Male	√	NA
Poly I:C	707				
		78	Female		Vehicle
		79	Female	√	Olanzapine
		80	Female	√	Risperidone
		81	Female		Risperidone
		434	Male	√	NA
		435	Male	√	NA
		436	Male	√	NA
Poly I:C	708				
		82	Female	√	Vehicle
		83	Female		Olanzapine
		84	Female		Risperidone
		437	Male	√	NA
		438	Male	√	NA

Note. This table lists each individual offspring (RAT_ID) with its litter identifier (DAM_ID), prenatal treatment, sex, and postnatal drug assignment. All linear mixed models included DAM_ID as a random intercept to account for the hierarchical data structure and within-litter correlation.

Column definitions:

Prenatal Treatment: Saline (control) or Poly I:C (MIA).

Dam ID: Litter identifier; littermates share the same ID.

RAT_ID: Unique offspring identifier.

Sex: Male / Female.

PD60_Blood: Randomly selected for blood sampling at postnatal day 60 to assess metabolic parameters (√ = sampled; blank = not sampled).

PD70_Drug (females only): Antipsychotic treatment from PD70 (Vehicle, olanzapine, risperidone); males coded as NA (not applicable).

Supplementary Table 7. Linear mixed model results for PD60 metabolic outcomes: fixed effects of prenatal treatment, sex, and their interactions.

Outcome	Variable	*F*(df)	*p*
**TC**			
	Prenatal	$F(1,14.1) = 15.41$	$p = 0.002$
	Sex	$F(1,53.5) = 5.73$	$p = 0.020$
	Prenatal × Sex	$F(1,53.5) = 3.68$	$p = 0.060$
**TG**			
	Prenatal	$F(1,13.8) = 5.19$	$p = 0.039$
	Sex	$F(1,53.6) = 0.12$	$p = 0.731$
	Prenatal × Sex	$F(1,53.6) = 2.82$	$p = 0.099$
**HDL-c**			
	Prenatal	$F(1,14.3) = 7.12$	$p = 0.018$
	Sex	$F(1,53.6) = 1.74$	$p = 0.193$
	Prenatal × Sex	$F(1,53.6) = 10.3$	$p = 0.002$
**LDL-c**†			
	Prenatal	$F(1,14.2) = 4.02$	$p = 0.065$
	Sex	$F(1,53.7) = 14.16$	$p = 0.000$
	Prenatal × Sex	$F(1,53.7) = 0.00$	$p = 0.952$
**GLU**†			
	Prenatal	$F(1,67.0) = 3.62$	$p = 0.061$
	Sex	$F(1,67.0) = 8.41$	$p = 0.005$
	Prenatal × Sex	$F(1,67.0) = 0.45$	$p = 0.506$

Note:This table reports Type III tests from linear mixed models (LMMs) for PD60 outcomes. Fixed effects were Prenatal treatment (Saline *vs*. Poly I:C), Sex (Male *vs*. Female), and Prenatal × Sex interaction. A random intercept for Dam ID (litter) was included to account for within-litter correlation. F-statistics with degrees of freedom, two-sided p-values, and (where applicable) partial eta-squared (η^2) are presented. To satisfy normality assumptions, outcomes with skewed residuals were analyzed after natural log (ln) transformation; for these outcomes, all statistics in this table are reported on the ln (model) scale and are not back-transformed. Primary analyses included all observations without outlier removal; outlier-excluded results are provided for sensitivity analysis (Tables S8–S9).

Supplementary Table 8. PD60 outlier counts (ROUT, Q = 1%) by outcome, prenatal condition, and Sex.

Outcome	Outlier detection scale (ROUT input)	Poly I:C + Female (N = 15)	Saline + Female (N = 15)	Poly I:C + Male (N = 18)	Saline + Male (N = 23)	Total outliers (n/N)
TC	Raw	-	-	-	-	-
TG	Raw	-	-	-	-	-
HDL-c	Raw	1	-	-	-	1/71
LDL-c†	In(x)	-	2	1	-	3/71
GLU†	In(x)	-	1	-	-	1/71

Note:Outlier identification was performed only for sensitivity analyses. The ROUT method (Q=1%) was implemented in GraphPad Prism on the analysis scale indicated (Raw or ln). Each cell reports the number of outliers identified in that subgroup; the total column gives outliers / total N for each outcome. Primary analyses did not remove outliers; outlier-excluded results are provided for robustness checks (see Table S9).

Supplementary Table 9. PD60 sensitivity analysis for outliers: comparison of primary and ROUT-excluded LMM results.

**Outcome**	**Variable**	**Primary *F*(df)**	**Primary *p***	**Sensitivity *F*(df)**	**Sensitivity *p***	**change?**
**HDL-c**						NO
	Prenatal	7.12(1,14.3)	$p = 0.018$	8.27(1,14.4)	$p = 0.012$	
	Sex	1.74(1,53.6)	$p = 0.193$	0.99(1,53.1)	$p = 0.326$	
	Prenatal × Sex	10.30(1,53.6)	$p = 0.002$	8.48(1,53.1)	$p = 0.005$	
**LDL-c†**						NO
	Prenatal	4.02(1,14.2)	$p = 0.065$	2.03(1,13.8)	$p = 0.176$	
	Sex	14.16(1,53.7)	$p < 0.001$	28.13(1,52.1)	$p < 0.001$	
	Prenatal × Sex	0.00(1,53.7)	$p = 0.952$	0.34(1,52.1)	$p = 0.564$	
**GLU†**						NO
	Prenatal	3.62(1,67.0)	$p = 0.061$	2.33(1,66)	$p = 0.131$	
	Sex	8.41(1,67.0)	$p = 0.005$	6.80(1,66)	$p = 0.011$	
	Prenatal × Sex	0.45(1,67.0)	$p = 0.506$	0.04(1,66)	$p = 0.847$	

Note: This table compares the primary analysis (all observations retained) with a sensitivity analysis excluding ROUT-identified outliers under the same LMM specification (fixed: Prenatal, Sex, Prenatal × Sex; random: Dam ID). For outcomes analyzed after ln transformation, statistics are reported on the ln (model) scale. The “Change” column indicates any substantial change from the primary analysis (e.g., change in statistical significance at α = 0.05 or reversal of effect direction); "NO" indicates no substantive change. Primary conclusions are based on all-data analyses, with outlier exclusion performed solely for sensitivity checks.

Supplementary Table 10. Linear mixed model results for PD70 metabolic outcomes: fixed effects of prenatal treatment, drug, and their interaction.

Outcome	Variable	*F*(df)	*p*
**TC**			
	Prenatal	$F(1,12.44) = 1.26$	$p = 0.283$
	Drug	$F(2,50.01) = 5.63$	$p = 0.006$
	Prenatal × Drug	$F(2,50.01) = 1.30$	$p = 0.282$
**TG**[†]			
	Prenatal	$F(1,14.22) = 0.27$	$p = 0.613$
	Drug	$F(2,52.68) = 0.85$	$p = 0.435$
	Prenatal × Drug	$F(2,52.68) = 0.62$	$p = 0.544$
**HDL-c**			
	Prenatal	$F(1,12.18) = 0.89$	$p = 0.363$
	Drug	$F(2,50.02) = 5.39$	$p = 0.008$
	Prenatal × Drug	$F(2,50.02) = 1.32$	$p = 0.276$
**LDL-c**			
	Prenatal	$F(1,12.74) = 6.17$	$p = 0.028$
	Drug	$F(2,51.31) = 0.84$	$p = 0.437$
	Prenatal × Drug	$F(2,51.31) = 0.41$	$p = 0.663$
**GLU**			
	Prenatal	$F(1,14.56) = 0.33$	$p = 0.576$
	Drug	$F(2,52.46) = 5.23$	$p = 0.008$
	Prenatal × Drug	$F(2,52.46) = 0.08$	$p = 0.922$
**Insulin**			
	Prenatal	$F(1,12.49) = 0.72$	$p = 0.414$
	Drug	$F(2,53.26) = 5.88$	$p = 0.005$
	Prenatal × Drug	$F(2,53.26) = 3.08$	$p = 0.054$
**HOMA-IR**[†]			
	Prenatal	$F(1,13.29) = 1.03$	$p = 0.328$
	Drug	$F(2,52.65) = 7.23$	$p = 0.002$
	Prenatal × Drug	$F(2,52.65) = 3.58$	$p = 0.035$
**Food efficiency**			
	Prenatal	$F(1,64.00) = 1.34$	$p = 0.251$
	Drug	$F(2,64.00) = 11.12$	$p < 0.001$
	Prenatal × Drug	$F(2,64.00) = 0.33$	$p = 0.722$
**Body weight gain**			
	Prenatal	$F(1,64.00) = 0.96$	$p = 0.332$

Outcome	Variable	*F*(df)	*p*
	Drug	$F(2,64.00) = 13.82$	$p < 0.001$
	Prenatal × Drug	$F(2,64.00) = 0.27$	$p = 0.761$
**Body length**			
	Prenatal	$F(1,14.28) = 1.48$	$p = 0.243$
	Drug	$F(2,53.41) = 0.32$	$p = 0.727$
	Prenatal × Drug	$F(2,53.41) = 0.38$	$p = 0.684$
**BMI**			
	Prenatal	$F(1,64.00) = 0.02$	$p = 0.896$
	Drug	$F(2,64.00) = 5.72$	$p = 0.005$
	Prenatal × Drug	$F(2,64.00) = 0.09$	$p = 0.916$
**Total white fat**			
	Prenatal	$F(1,13.07) = 2.04$	$p = 0.177$
	Drug	$F(2,53.53) = 27.61$	$p < 0.001$
	Prenatal × Drug	$F(2,53.53) = 0.75$	$p = 0.479$
**Water intake**			
	Prenatal	$F(1,64.00) = 0.70$	$p = 0.406$
	Drug	$F(2,64.00) = 5.74$	$p = 0.005$
	Prenatal × Drug	$F(2,64.00) = 1.76$	$p = 0.181$
**Food intake**			
	Prenatal	$F(1,64.00) = 0.11$	$p = 0.739$
	Drug	$F(2,64.00) = 9.64$	$p < 0.001$
	Prenatal × Drug	$F(2,64.00) = 0.06$	$p = 0.938$
**24 h wheel running- total distance**			
	Prenatal	$F(1,64.00) = 7.25$	$p = 0.009$
	Drug	$F(2,64.00) = 8.43$	$p = 0.001$
	Prenatal × Drug	$F(2,64.00) = 5.88$	$p = 0.005$
**24 h wheel running- total distance of night**			
	Prenatal	$F(1,14.99) = 0.00$	$p = 0.994$
	Drug	$F(2,53.19) = 11.07$	$p < 0.001$
	Prenatal × Drug	$F(2,53.19) = 2.65$	$p = 0.080$
**Prolactin**			
	Prenatal	$F(1,63.00) = 0.00$	$p = 0.952$
	Drug	$F(2,63.00) = 128.33$	$p < 0.001$
	Prenatal × Drug	$F(2,63.00) = 0.93$	$p = 0.400$
**Adiponectin**†			

Outcome	Variable	*F*(df)	*p*
	Prenatal	$F(1,12.39) = 8.92$	$p = 0.011$
	Drug	$F(2,50.92) = 8.25$	$p = 0.001$
	Prenatal × Drug	$F(2,50.92) = 1.35$	$p = 0.269$
**Leptin**†			
	Prenatal	$F(1,13.28) = 5.03$	$p = 0.043$
	Drug	$F(2,51.70) = 17.88$	$p < 0.001$
	Prenatal × Drug	$F(2,51.70) = 1.76$	$p = 0.183$

Note: This table reports Type III tests from linear mixed models (LMMs) for PD70 outcomes. Fixed effects were Prenatal (Saline *vs*. Poly I:C), Drug (Vehicle, Olanzapine, Risperidone), and Prenatal × Drug. A random intercept for Dam ID (litter) was included to account for within-litter correlation. F-statistics with degrees of freedom, two-sided p-values, and partial eta-squared (η^2) are shown. For outcomes analyzed after natural log (ln) transformation, all statistics in this table are reported on the ln (model) scale and are NOT back-transformed. Primary analyses retained all observations; outlier-excluded results are provided as sensitivity checks (Tables S11–S12). Degrees of freedom were estimated using the Satterthwaite approximation.

Supplementary Table 11. PD70 outlier counts (ROUT, Q = 1%) by outcome, prenatal condition, and drug treatment.

Outcome	Saline + Veh (N = 12)	Saline + OLZ (N = 12)	Saline + RISP (N = 12)	Poly I:C+ Veh (N = 11)	Poly I:C+ OLZ (N = 11)	Poly I:C+ RISP (N = 12)	Total outliers (n/N)
TC	-	-	-	-	1	-	1/70
TG†	-	-	-	-	-	-	-
HDL-c	-	-	-	-	-	-	-
LDL-c	-	-	-	-	-	-	-
GLU	-	-	-	-	1	-	1/70
Insulin†	-	-	-	-	-	-	-
HOMA-IR†	-	-	-	-	-	-	-
Food efficiency	-	-	-	-	-	-	-
Body weight gain	-	-	-	-	-	-	-
Body length	-	-	-	-	-	-	-
BMI	-	-	-	-	1	-	1/70
Total white fat	1	-	-	-	-	-	1/70
Water intake	-	-	-	-	-	-	-
Food intake	-	-	-	-	-	-	-
24 h wheel running- total distance	-	-	-	-	-	1	1/70
24 h wheel running- total distance of night	-	1	-	-	1	-	2/70
Prolactin	-	-	1	-	1	-	2/70
Adiponectin†	-	-	-	-	-	-	-
Leptin†	-	-	-	-	-	-	-
AUC	-	-	-	-	-	-	-

Note: Outlier identification was performed for sensitivity analyses only. ROUT (Q = 1%) was implemented in GraphPad Prism, using the scale indicated in the table (Raw or ln(x)). Each cell reports the number of identified outliers for that subgroup; the total column reports total outliers/total N for each outcome. Primary analyses did not remove outliers; outlier-excluded results are provided only as robustness checks (see Table S12). Veh, vehicle; OLZ, olanzapine; RISP, risperidone.

Supplementary Table 12. PD70 outlier-sensitivity analysis: primary (all data) *vs* outliers excluded (LMM).

Outcome	Variable	Primary *F*(df)	Primary *p*	Sensitivity *F*(df)	Sensitivity *p*	Change?
**TC**						NO
	Prenatal	1.26(1,12.44)	0.283	3.16(1,12.65)	0.100	
	Drug	5.63(2,50.01)	0.006	4.03(2,49.98)	0.024	
	Prenatal * Drug	1.30(2,50.01)	0.282	0.71(2,49.98)	0.495	
**GLU**						NO
	Prenatal	0.33(1,14.56)	0.576	0.08(1,15.23)	0.775	
	Drug	5.23(2,52.46)	0.008	7.43(2,52.49)	0.001	
	Prenatal * Drug	0.08(2,52.46)	0.922	0.48(2,52.49)	0.622	
**BMI**						NO
	Prenatal	0.02(1,64.00)	0.896	0.22(1,63.00)	0.642	
	Drug	5.72(2,64.00)	0.005	5.01(2,63.00)	0.010	
	Prenatal * Drug	0.09(2,64.00)	0.916	0.36(2,63.00)	0.698	
**Total white fat**						NO
	Prenatal	2.04(1,13.07)	0.177	1.62(1,63.00)	0.208	
	Drug	27.61(2,53.53)	0.000	29.81(2,63.00)	0.000	
	Prenatal * Drug	0.75(2,53.53)	0.479	0.87(2,63.00)	0.426	
**24 h wheel running-total distance**						NO
	Prenatal	7.25(1,64.00)	0.009	8.49(1,63.00)	0.005	
	Drug	8.43(2,64.00)	0.001	8.74(2,63.00)	0.000	
	Prenatal * Drug	5.88(2,64.00)	0.005	6.13(2,63.00)	0.004	
**24 h wheel running-total distance of night**						NO
	Prenatal	0.00(1,14.99)	0.994	0.01(1,15.41)	0.925	
	Drug	11.07(2,53.19)	0.000	10.32(2,51.92)	0.000	
	Prenatal * Drug	2.65(2,53.19)	0.080	3.10(2,51.92)	0.054	
**Prolectin**						NO
	Prenatal	0.00(1,63.00)	0.952	0.05(1,62.00)	0.832	
	Drug	128.33(2,63.00)	0.000	137.11(2,62.00)	0.000	
	Prenatal * Drug	0.93(2,63.00)	0.400	0.72(2,62.00)	0.490	

Note: This table compares the primary analysis (all observations retained) with a sensitivity analysis excluding

ROUT-identified outliers under the same LMM specification (fixed: Prenatal, Drug, Prenatal × Drug; random: Dam ID). For outcomes analyzed after ln transformation, statistics are reported on the ln (model) scale. The "Change?" column indicates whether the conclusion regarding statistical significance ($p < 0.05$) or effect direction changed after outlier exclusion; "NO" indicates no substantive change. Primary inference is based on the all-data analyses; outlier exclusion was performed only as a sensitivity check.
